# Immunological risk factors for sepsis-associated delirium and mortality in ICU patients

**DOI:** 10.3389/fimmu.2022.940779

**Published:** 2022-09-20

**Authors:** Wen Lei, Zhiyao Ren, Jun Su, Xinglong Zheng, Lijuan Gao, Yudai Xu, Jieping Deng, Chanchan Xiao, Shuai Sheng, Yu Cheng, Tianshun Ma, Yu Liu, Pengcheng Wang, Oscar Junhong Luo, Guobing Chen, Zhigang Wang

**Affiliations:** ^1^ Department of Microbiology and Immunology, School of Medicine, Jinan University, Guangzhou, China; ^2^ Institute of Geriatric Immunology, School of Medicine, Jinan University, Guangzhou, China; ^3^ Guangdong-Hong Kong-Macau Great Bay Area Geroscience Joint Laboratory, Jinan University, Guangzhou, China; ^4^ Department of Systems Biomedical Sciences, School of Medicine, Jinan University, Guangzhou, China; ^5^ National Health Commission (NHC) Key Laboratory of Male Reproduction and Genetics, Guangzhou, China; ^6^ Department of Central Laboratory, Guangdong Provincial Reproductive Science Institute (Guangdong Provincial Fertility Hospital), Guangzhou, China; ^7^ Department of Sonograph, The First Affiliated Hospital, Jinan University, Guangzhou, China; ^8^ Department of Critical Care Medicine, The First Affiliated Hospital, Jinan University, Guangzhou, China

**Keywords:** ICU, sepsis-associated delirium, immune profile, mortality, gene expression, monocyte

## Abstract

**Background:**

A major challenge in intervention of critical patients, especially sepsis-associated delirium (SAD) intervention, is the lack of predictive risk factors. As sepsis and SAD are heavily entangled with inflammatory and immunological processes, to identify the risk factors of SAD and mortality in the intensive care unit (ICU) and determine the underlying molecular mechanisms, the peripheral immune profiles of patients in the ICU were characterized.

**Methods:**

This study contains a cohort of 52 critical patients who were admitted to the ICU of the First Affiliated Hospital of Jinan University. Comorbidity, including sepsis and SAD, of this cohort was diagnosed and recorded. Furthermore, peripheral blood samples were collected on days 1, 3, and 5 of admission for peripheral immune profiling with blood routine examination, flow cytometry, ELISA, RNA-seq, and qPCR.

**Results:**

The patients with SAD had higher mortality during ICU admission and within 28 days of discharge. Compared with survivors, nonsurvivors had higher neutrophilic granulocyte percentage, higher CRP concentration, lower monocyte count, lower monocyte percentage, lower C3 complement level, higher CD14^lo^CD16^+^ monocytes percentage, and higher levels of IL-6 and TNFα. The CD14^hi^CD16^-^ monocyte percentage manifested favorable prediction values for the occurrence of SAD. Differentially expressed genes between the nonsurvival and survival groups were mainly associated with immune response and metabolism process. The longitudinal expression pattern of SLC2A1 and STIMATE were different between nonsurvivors and survivors, which were validated by qPCR.

**Conclusions:**

Nonsurvival critical patients have a distinct immune profile when compared with survival patients. CD14^hi^CD16^-^ monocyte prevalence and expression levels of SLC2A1 and STIMATE may be predictors of SAD and 28-day mortality in ICU patients.

## Introduction

The intensive care unit (ICU) admits heterogeneous patients with life-threatening conditions, leading to high mortality. To reduce the mortality rate in the ICU, it is important to identify and predict the course of death, which may also be critical for the diagnosis, intervention, and prognosis of patients. Risk stratification predictive factors can provide useful information for physicians and help improve clinical decision making. Existing risk stratification methods based on commonly measured clinical and physiologic parameters have played important roles in risk prediction and prognostication of patients in the ICU (1).

Sepsis, one of the leading causes of death in the ICU, is a complex clinical syndrome resulting from a systemic, dysregulated inflammatory response to uncontrolled infections (2). Many ICU patients with sepsis also develop neurological defects, known as sepsis-associated encephalopathy (SAE), including delirium, as part of multiple organ failure. Delirium is the main early symptom of SAE, which is also known as acute encephalopathy syndrome (3). It is difficult to control sepsis-associated delirium (SAD) once it emerges; therefore, it is important to make an early diagnosis and start treatment. Effective auxiliary indicators or predictive factors are needed to aid the early diagnosis of SAD and to ultimately prevent death in the ICU.

Clinically, a multitude of measures have been designed to assess and screen delirium. Currently used delirium severity measures, such as the Delirium Rating Scale-Revised-98 (DRS-R-98) (4) or the Memorial Delirium Assessment Scale (MDAS) (5), provide mixed coverage of delirium signs and symptoms to psychiatrists. Moreover, multiple tools have been developed and validated to help nonpsychiatrists screen possible delirium. The Confusion Assessment Method–Intensive Care Unit (CAM-ICU) and the Intensive Care Delirium Screening Checklist (ICDSC) are the two tools that are recommended for diagnosing delirium in the ICU by the Pain, Agitation/Sedation, Delirium, Immobility, and Sleep Disruption in Adult Patients in the ICU (PADIS) guidelines (6, 7). In addition, electroencephalogram (EEG) is an adjunctive biomarker to assess for delirium, and it needs to combine with a clinical assessment for the presence of delirium symptoms (8). Thus, mastering delirium pathophysiology, understanding the association of delirium with outcomes, and further understanding delirium biomarkers and their practical use in predictive models are needed for improved delirium diagnosis and prognosis (9).

For critical patients, dysregulated immune response is one of the key factors for determining the severity of disease progression and prognosis (10–12). For instance, sepsis occurs when the body’s immune system overreacts to infection and causes damage to its own tissues and organs (13). Therefore, monitoring the state of the immune system can help clinicians to early assess the risk of death for critical patients. In addition, understanding the immunological characteristics and the underlying molecular mechanism not only aids the diagnosis but also can help in developing novel treatment methods for critical patients.

To this end, we recruited a cohort of ICU patients and used laboratory methods to characterize their peripheral immune features longitudinally in an effort to identify potential immunological factors for the early diagnosis of SAD and patient outcome prediction. We utilized immune and genetic laboratory assays from three time points after ICU admission to evaluate immunological risk factors as related to the presence of delirium and mortality in sepsis patients.

## Materials and methods

### Experimental design

This single-center prospective and longitudinal study was conducted in a 12-bed ICU ward of the First Affiliated Hospital of Jinan University. This study was approved by the Ethics Committee at the First Affiliated Hospital of Jinan University (Guangzhou, China) (KY-2020-004). Each patient or the patient’s family member signed the informed consent form before participation. All procedures performed in studies involving human participants were in accordance with the ethical standards of the ethics committee and with the 1964 Declaration of Helsinki and its later amendments or comparable ethical standards.

### Patients

Adult patients who were consecutively admitted to the ICU between July and November 2019 were enrolled. Patients meeting the following criteria were excluded: age <18 years or >85 years, stayed in ICU fewer than 3 days, traumatic brain injury, intracerebral hemorrhage, cerebral infarction, immunological diseases, malignant hematologic diseases, drug abuse, severe circulatory instability, allergic constitution, terminal state, or pregnancy. After removing the excluded patients, a total of 52 patients were retained.

### Clinical data collection

The following demographic and clinical data were collected:

1. Demographic characteristics: age, gender.
2. Clinical scores: Acute Physiology and Chronic Health Evaluation (APACHE) II scores.
3. Comorbidity: coronary heart disease (CHD), chronic obstructive pulmonary disease (COPD), chronic renal failure (CRF), liver cirrhosis, hypertension, diabetes, sepsis, delirium. Sepsis was clinically diagnosed according to the Third International Consensus Definitions for Sepsis and Septic Shock (Sepsis-3) (13). Delirium was clinically diagnosed by CAM-ICU (7). All assessments were performed by the ICU physicians of the First Affiliated Hospital of Jinan University.
4. Follow-up data: death occurring within ICU was recorded, and a follow-up was conducted for all patients until 28 days after ICU discharge.
5. Clinical information was also collected: length of stay in ICU, use of mechanical ventilation, ventilation time.

### Laboratory data collection

On days 1, 3, and 5 after ICU admission, peripheral blood samples were collected from patients for further laboratory assessments. Blood routine examination and flow cytometry were tested for whole blood. Serum was isolated for enzyme-linked immunosorbent assay (ELISA) analysis, and PBMC cells were isolated for RNA sequencing and quantitative real-time polymerase chain reaction (qPCR) detection.

### Blood routine examination

Blood routine examination (white blood cell count, neutrophil percent, lymphocyte percent, monocyte percent, eosinophil percent, neutrophil count, lymphocyte count, monocyte count, platelet count, CRP concentration, C3 concentration, C4 concentration, IgA concentration, IgG concentration, IgM concentration, κ concentration, λ concentration, and κ/λ) was performed by an automatic blood cell analyzer.

### Flow cytometry detection

Antibodies of flow cytometry detection were from Biolegend, and NovoCyte 2060R (Agilent) was used for analysis. Identification of immune cell subsets as T lymphocytes (CD3^+^CD19^-^), B lymphocytes (CD3^-^CD19^-^), classical monocyte cell (CD3^-^CD19^-^CD14^hi^CD16^-^), intermediate monocytes (CD3^-^CD19^-^CD14^hi^CD16^+^), nonclassical monocytes (CD3^-^CD19^-^CD14^lo^CD16^+^), and natural killer cell (CD3^-^CD19^-^CD14^-^CD16^+^CD56^+^) staining was performed with the following antibodies: CD3-FITC (UCHT1), CD19-PerCP/Cy5.5 (HIB19), CD14-PE/Cy7 (63D3), CD16-APC (3G8), and CD56-PE (MEM-188).

### ELISA detection

The serum of all blood samples was isolated and stored at -80°C. IL6, IL10, and TNF-α were determined with ELISA kits (ABclonal) following the manufacturer’s manual.

### RNA extraction, sequencing, and analysis

PBMCs were isolated from the blood of healthy donors using Ficoll (GE Healthcare Life Sciences) following the manufacturer’s protocol. Total RNA was isolated by a TRIzol Reagent (Invitrogen). The library preparations were sequenced on an Illumina Novaseq platform, and 150-bp paired-end reads were generated.

Differential expression analysis of the survival and nonsurvival groups was performed using the DESeq2 R package (1.30.1). Gene Ontology (GO) and Kyoto Encyclopedia of Genes and Genomes (KEGG) enrichment analysis was performed with the topGO R package (2.42.0). The DE genes clustered with k-means clustering using the *z*-score transformed expression levels. Gene set enrichment analysis (GSEA) was performed with the clusterProfiler R package (3.18.1). The KEGG data set (v 7.4) was used for the enrichment input of GSEA independently (http://www.gsea-msigdb.org/gsea/).

### Quantitative real-time polymerase chain reaction analysis

Total RNA from PBMCs was extracted with TRIzol (Life Technologies), and the first-strand cDNA was synthesized by an ABscript II cDNA First Strand Synthesis Kit (ABclonal) according to the manufacturer’s protocol. qPCR was performed using 2X Universal SYBR Green Fast qPCR Mix (ABclonal) in the CFX Connect Real-Time System (BIO-RAD).

### Statistical analysis

Normally distributed continuous variables are reported using mean and SD, whereas abnormally distributed continuous variables are reported as median and interquartile range (IQR). Categorical variables use the number of observations and percentages. Factors associated with mortality were explored in univariate analysis using Student’s *t*-test or the Mann*–*Whitney U test for continuous variables and Pearson’s chi-square or Fisher’s exact test for categorical variables as appropriate. Factors associated with mortality in univariate analysis (*p* <.1) were entered in a multivariate regression. Five clinical indexes (age, APACH II, diabetes, sepsis, and delirium) and 11 lab analysis indexes (neutrophil percent, monocyte percent, monocyte count, CRP concentration, C3 concentration, CD14^hi^CD16^-^ monocyte percent, CD14^lo^CD16^+^ monocyte percent, IL6 concentration, TNFα concentration, SLC2A1, and STIMATE) were entered. These indices are summarized in **Table S9**. Before multivariate regression analysis, missing values were completed by the mice R package (3.13.0). Then, multiple linear regression was analyzed by the leaps R package (3.1). This is a single linear model regression analysis for multiple variates, i.e., the clinical and lab test variables together fitting toward the patient outcome (dead or alive) rather than multiple regression tests; therefore, Bonferroni correction for multiple comparisons was not used. A two-sided *p*-value of.05 was considered statistically significant. All analysis and data plotting were performed by SPSS 22.0 statistical software, R Statistical Software, or GraphPad Prism 7.00. Fluorescence-activated cell sorting (FACS) of the data was performed by FLOWJO 10 software.

## Results

### Cohort clinical characteristics

In this study, we recruited a cohort of patients admitted to the ICU to explore the immunological risk factors of SAD and mortality. In total, 63 patients were recruited during July to December 2019, and 11 patients were excluded. Consequently, 52 patients were enrolled in the study (the Examinational cohort). In the Examinational cohort (**Table 1**), the mean age of the enrolled patients was 64.48 (SD = 14.47), and 63.46% of the patients were men. The median APACHE II score was 19.5 (IQR 15.25–26), and 48.1% of the patients were treated by mechanical ventilation (MV). The mean ICU admission time was 8.38 days (SD = 9.40). For comorbidity, 53.9%, 17.31%, 5.77%, 23.08%, 1.92%, 34.6%, and 32.7% of the patients suffered from sepsis, CHD, COPD, CRF, liver cirrhosis, hypertension, and diabetes, respectively. For all the enrolled patients, the overall survival after treatment was examined for 28 days. The results show that 10 (19.23%) patients died. Between the survival and nonsurvival groups, there was significant difference in age (*p* = .015), sepsis (*p* = .028), and delirium (*p* = .006).

**Table 1:** Baseline characteristics of examinational cohort and subset cohort of sepsis.

Cohort	Examinational cohort				Subset cohort of sepsis			
Baseline characteristics	All patients	Nonsurvival	Survival	p-value	Sepsis patients	Nondelirium	Delirium	p-value
Number	52	10	42		28	11	17	
Gender (men/women)	33/19	7/3	26/16	0.910^b^	19/9	7/4	12/5	1.000^b^
Age mean ± SD [y]	64.48 ± 14.47	73.30 ± 10.70	62.38 ± 14.56	0.015^c^	65.00 ± 14.07	56.36 ± 16.81	70.59 ± 8.56	0.022^c^
APACHE II score median (IQR)	19.5 (15.25–26)	24 (17.75–29.5)	18.5 (15–25.25)	0.116^a^	21 (15–25)	15 (12–20)	24 (18–28)	0.005^a^
ICU days mean ± SD [d]	8.38 ± 9.40	10.50 ± 6.92	7.88 ± 9.91	0.434^c^	10.50 ± 12.12	5.55 ± 2.34	13.71 ± 14.71	0.038^c^
Mechanical ventilation (%)	25 (48.1%)	7 (70%)	18 (42.9%)	0.233^b^	14 (50%)	4 (36.4%)	10 (58.8%)	0.440^b^
Ventilation time median (IQR) [h]	0 (0–67.75)	54 (0–252.5)	0 (0–41.75)	0.080^a^	14 (0–85.25)	0 (0–31.5)	42 (0–101)	0.264^a^
Death (%)	**-**				9 (32.1%)	1 (9.1%)	8 (47.1%)	0.049^b^
Comorbidity(%)								
Sepsis	28 (53.9%)	9 (90%)	19 (25.2%)	0.028^b^	**-**			
Delirium	24 (46.2%)	9 (90%)	15 (35.7%)	0.006^b^	**-**			
CHD	9 (17.31%)	3 (30%)	6 (14.29%)	0.474^b^	8 (28.6%)	3 (27.3%)	5 (29.4%)	0.624^b^
COPD	3 (5.77%)	1 (10%)	2 (4.76%)	1.000^b^	3 (10.7%)	1 (9.1%)	2 (11.8%)	0.664^b^
CRF	12 (23.08%)	3 (30%)	9 (21.43%)	0.872^b^	7 (25%)	3 (27.3%)	4 (23.5%)	0.581^b^
Liver cirrhosis	1 (1.92%)	0 (0%)	1 (2.38%)	1.000^b^	0 (0%)	0 (0%)	0 (0%)	–
Hypertension	18 (34.6%)	6 (60%)	12 (28.6%)	0.132^b^	12 (42.9%)	6 (54.5%)	6 (35.3%)	0.441^b^
Diabetes	17 (32.7%)	6 (60%)	11 (26.2%)	0.094^b^	11 (39.3%)	5 (45.5%)	6 (35.3%)	0.701^b^

APACHE II, Acute Physiology and Chronic Health Evaluation II; ICU, intensive care unit; IQR, interquartile range; CHD, coronary heart disease; COPD, chronic obstructive pulmonary disease; CRF, chronic renal failure.

a Mann–Whitney U test comparing 28-day survivors with nonsurvivors or delirium patients with nondelirium patients.

b χ^2^ test comparing 28-day survivors with nonsurvivors or delirium patients with nondelirium patients (Fisher’s exact test, two-sided).

c t-test comparing 28-day survivors with nonsurvivors or delirium patients with nondelirium patients.

The percentages in columns are noted as within group by column.

### Peripheral immune profiling of ICU patients

In order to identify potential clinical and molecular predictors of SAD and its mortality in patients admitted to the ICU, the blood samples of the Examinational cohort were collected sequentially on days 1, 3, and 5 for a series of tests.

**Figure 1:**
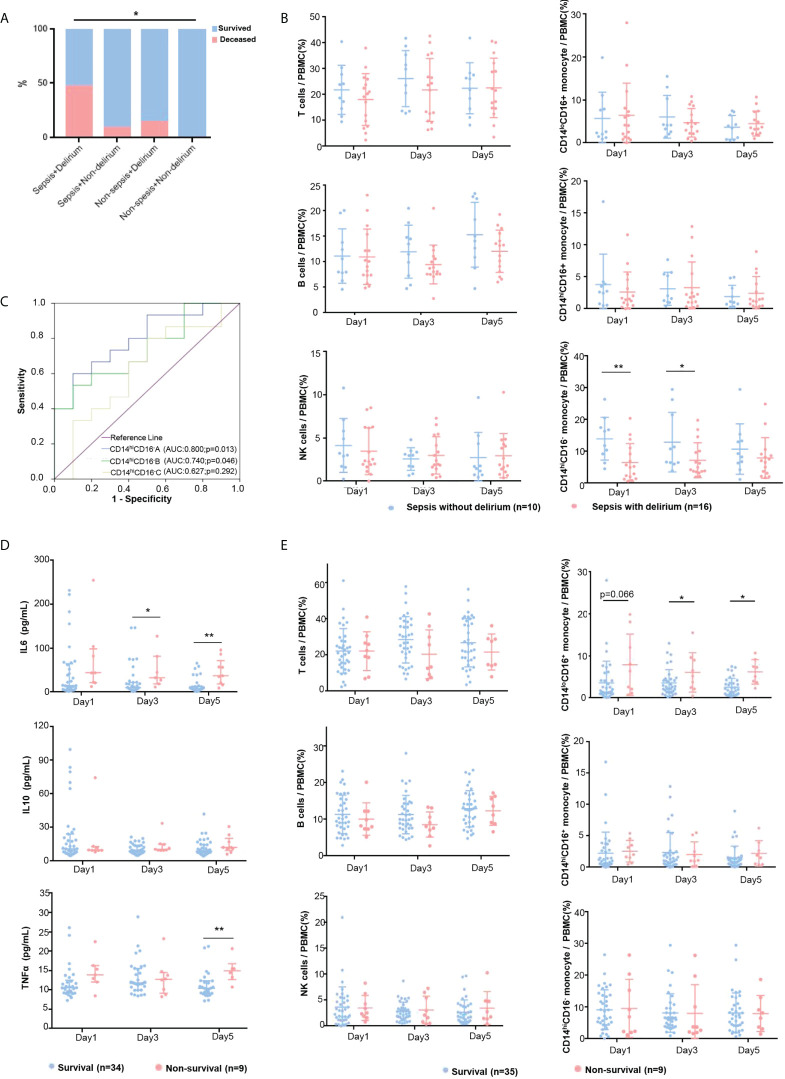
Peripheral immune profiling of critical patients. **(A)** Survival rate of eligible ICU patients of examinational cohort. The mortality rates of four groups were compared using Pearson’s chi-square test. **(B)** The immune cell proportions in patients with SAD and patients with sepsis were compared at days 1, 3, and 5. The patients with SAD (red dots) and sepsis patients without delirium (blue dots) were compared by *t*-test. Plots show mean and SD. **(C)** ROC curves for CD14^hi^CD16^-^ monocytes at days 1, 3, and 5 for predicting the incidence of delirium in sepsis patients. **(D)** The levels of plasma cytokines were tested in nonsurvival and survival groups with ELISA at days 1, 3, and 5. The 28-day survivors (blue dots) and nonsurvivors (red dot) were compared by the nonparametric Mann–Whitney U test. Plots show median and IQR. **(E)** Immune cell proportions in 28-day nonsurvivors and survivors were compared on admission days 1, 3, and 5. The 28-day survivors (blue dots) and nonsurvivors (red dots) were compared by *t*-test. Plots show mean and SD. * refers to *p* <.05, and ** refers to *p* <.01.

Sepsis and delirium were the risk factors critically affecting the mortality of ICU patients, especially when sepsis is complicated with delirium. More than half of the 52 patients within the Examinational cohort had sepsis (*n* = 28, 53.9%), and 17 (60.7%) of these 28 sepsis patients had delirium (**Table 1**). Eight (47.1%) of these 17 patients with sepsis and delirium died during ICU admission or within 28 days of discharge, which was a significantly higher mortality rate than the rest (**Figure 1A**). Among the sepsis patients, the association between delirium and the risk for mortality was weakly significant (nondelirium vs. delirium: 9.1% vs. 47.1%, *p* = .049) (**Table 1**). In other words, ICU patients with both sepsis and delirium are much more likely to develop an unfavorable outcome (death).

**Figure 2:**
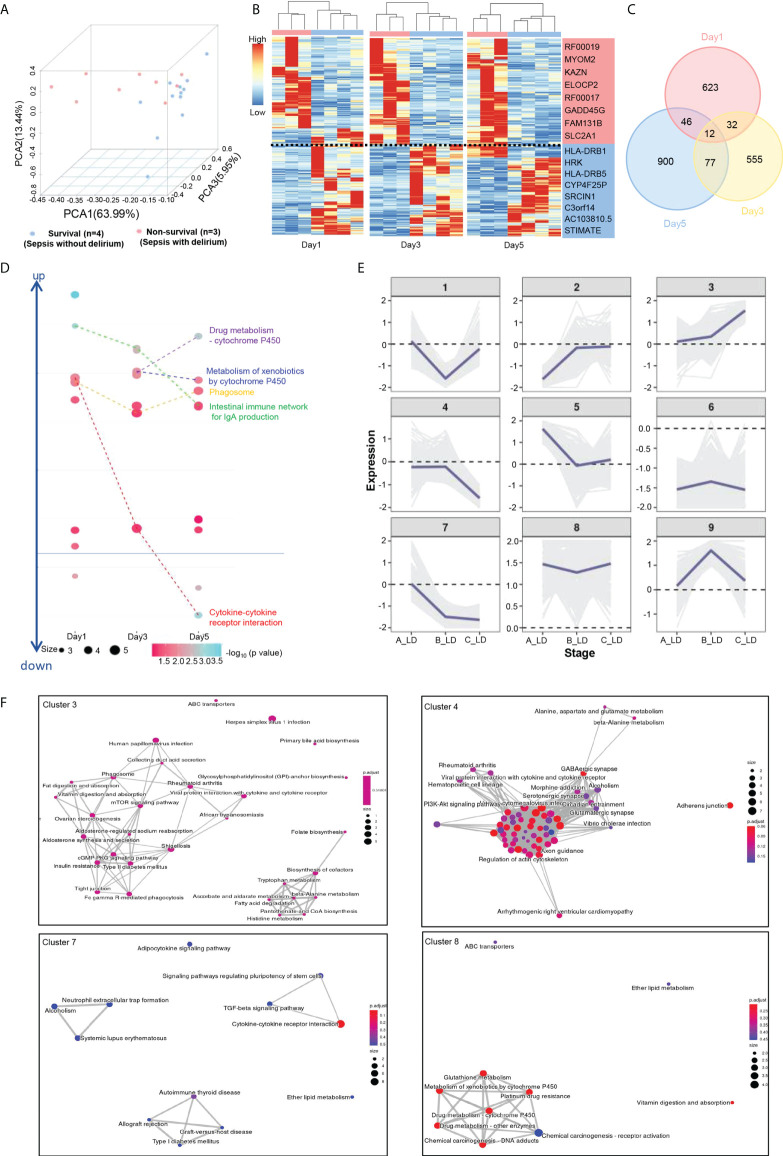
Transcriptional signatures of SAD and K-means analysis of DE genes. **(A)** PCA plot for RNA-seq data. All survival samples (blue dots) and nonsurvival samples (red dots) are shown. **(B)** Heat map visualization of DE genes (*p* value <.05 and fold change ≥ 2) between survival (blue) and nonsurvival (red) patients. **(C)** Venn diagram of DE genes between survival and nonsurvival groups. **(D)** The significantly enriched KEGG pathway for each group submitted DE genes are shown at days 1, 3, and 5. **(E)** Expression trends of DE genes from days 1–5. Genes were grouped as nine clusters with k-means clustering according to their expression patterns. The expression of each gene was calculated by normalized counts with the formula as follows. *Exp*_(per gene)_ = log2 (AVG- livability/AVG- mortality). *Exp*_(per gene)_: the expression value of each gene; AVG-mortality: average gene expression of nonsurvival; AVG-livability: average gene expression of survival. **(F)** The significantly enriched KEGG pathway for clusters 3, 4, 7, and 8.

The occurrence of delirium in sepsis patients was associated with age (*p* = .022) and APACHE II score (*p* = .005) (**Table 1**), and ICU stay in days of patients with SAD was significantly longer than that of septic patients without delirium (*p* = .038) (**Table 1**). There was no significant difference in gender, MV, and comorbidity (**Table 1**). FACS results revealed that patients who had sepsis complicated with delirium had lower CD14^hi^CD16^—^ monocyte prevalence (**Figure 1B** and **Table S1**). To test this further, we constructed ROC curves to determine the sensitivity and specificity of the CD14^hi^CD16^—^ monocyte percentage to predict the occurrence of delirium in sepsis patients. The areas under the curve (AUCs) for days 1, 3, and 5 were 0.800, 0.740, and 0.627, respectively (**Figure 1C**). The optimal cutoff for the CD14^hi^CD16^-^ monocyte percentage predictive value was 6.03% (day 1) and 5.52% (day 3), which yielded a sensitivity and specificity of 60.0% and 90.0% (day 1) and 53.3% and 90.0% (day 3), respectively, for predicting the occurrence of delirium in sepsis patients. These results suggest that ICU patients with sepsis have a higher likelihood of developing delirium if their blood CD14^hi^CD16^—^ monocyte prevalence is low.

Furthermore, we analyzed the differences of the peripheral immune profiling between survivors (*n* = 42) and nonsurvivors (*n* = 10) within the Examinational cohort. Blood routine examination (**Table 2**) showed that nonsurvivors had higher neutrophilic granulocyte percentage, higher CRP concentration, lower monocyte count, and lower monocyte percentage. There were no significant differences in the levels of complement and immunoglobulin except for the C3 complement. FACS results showed that a higher CD14^lo^CD16^+^ monocyte percentage was significantly associated with mortality at multiple time points (**Figure 1D**, **Table S2**). The patients who died within 28 days had higher levels of IL-6 and TNF-α compared with survivors, which is possibly related to dysregulated immune responses, whereas no clear difference in IL-10 was observed (**Figure 1E**, **Table S2**).

**Table 2:** Clinical and immune related biochemical data of nonsurvivors and survivors in examinational cohort.

Parameter	Survival	Nonsurvival	p -value^a^
**WBC day1 [×10^9^/L]**	11.01 (8.31–17.65)	8.21 (4.19–19.45)	0.816
**WBC day3 [×10^9^/L]**	11.10 (7.91–18.03)	12.51 (6.17–17.16)	0.871
**WBC day5 [×10^9^/L]**	10.38 (8.08–15.68)	10.82 (5.47–15.39)	0.553
**NEUT day1 [×10^9^/L]**	8.96 (6.93–15.77)	6.95 (3.56–18.47)	0.676
**NEUT day3 [×10^9^/L]**	9.15 (5.19–16.67)	11.46 (5.38–15.91)	0.531
**NEUT day5 [×10^9^/L]**	9.07 (5.76–14.03)	9.46 (4.71–14.08)	0.286
**NEUT% day1 [%]**	87.50 (81.70–92.40)	86.80 (79.95–94.80)	0.745
**NEUT% day3 [%]**	86.90 (78.80–90.60)	90.50 (85.36–94.73)	0.070
**NEUT% day5 [%]**	84.70 (75.90–88.10)	91.25 (83.25–91.25)	0.027
**LYMPH day1 [×10^9^/L]**	0.65 (0.40–0.98)	0.60 (0.54–0.74)	0.972
**LYMPH day3 [×10^9^/L]**	0.82 (0.63–1.36)	0.58 (0.48–0.91)	0.270
**LYMPH day5 [×10^9^/L]**	1.02 (0.61–1.33)	0.57 (0.38–0.79)	0.240
**LYMPH% day1 [%]**	6.00 (3.40–10.30)	8.65 (2.98–14.68)	0.898
**LYMPH% day3 [%]**	7.80 (4.80–12.20)	4.90 (3.18–11.83)	0.227
**LYMPH% day5 [%]**	9.30 (4.31–13.71)	5.05 (3.83–9.60)	0.155
**MONO day1 [×10^9^/L]**	0.66 (0.42–0.85)	0.36 (0.09–0.49)	0.128
**MONO day3 [×10^9^/L]**	0.62 (0.49–0.96)	0.27 (0.21–0.49)	0.006
**MONO day5 [×10^9^/L]**	0.67 (0.36–1.00)	0.45 (0.27–0.64)	0.079
**MONO% day1 [%]**	5.60 (3.90–8.70)	2.30 (1.80–5.65)	0.131
**MONO% day3 [%]**	5.70 (4.10–8.30)	2.85 (2.10–3.83)	0.002
**MONO% day5 [%]**	6.10 (5.40–7.90)	4.10 (2.98–5.25)	0.010
**PLT day1 [×10^9^/L]**	213.00 (116.20–307.00)	128.50 (12.00–191.50)	0.198
**PLT day3 [×10^9^/L]**	197.00 (120.00–257.00)	109.50 (58.75–184.50)	0.150
**PLT day5 [×10^9^/L]**	166.20 (92.00–262.00)	103.00 (42.50–177.25)	0.176
**CRP day1 [mg/L]**	76.57 (37.54–102.63)	195.30 (139.78–256.49)	0.001
**CRP day3 [mg/L]**	87.94 (41.87–139.79)	243.17 (204.65–300.76)	0.006
**CRP day5 [mg/L]**	55.56 (24.47–117.24)	211.74 (162.39–229.40)	0.001
**C3 day1 [mg/L]**	952.00 (820.00–1102.00)	708.50 (506.45–936.75)	0.035
**C3 day3 [mg/L]**	963.00 (837.00–1112.00)	734.50 (541.60–903.75)	0.006
**C3 day5 [mg/L]**	868.00 (806.00–1156.00)	774.50 (594.90–995.75)	0.442
**C4 day1 [mg/L]**	245.00 (214.00–354.00)	208.79 (157.00–272.00)	0.165
**C4 day3 [mg/L]**	237.00 (207.00–325.00)	209.19 (166.75–257.50)	0.046
**C4 day5 [mg/L]**	227.00 (181.00–363.00)	202.79 (156.75–267.25)	0.425
**IgA day1 [g/L]**	1.77 (1.36–2.85)	2.47 (1.88–3.91)	0.350
**IgA day3 [g/L]**	1.94 (1.46–2.50)	2.54 (2.19–3.89)	0.141
**IgA day5 [g/L]**	2.22 (1.46–2.56)	3.01 (2.36–3.94)	0.131
**IgG day1 [g/L]**	11.85 (9.01–15.12)	10.72 (8.75–16.44)	0.274
**IgG day3 [g/L]**	11.66 (9.05–14.96)	11.40 (8.38–18.00)	0.319
**IgG day5 [g/L]**	12.48 (8.38–16.17)	12.42 (8.82–20.83)	0.511
**IgM day1 [g/L]**	0.59 (0.46–0.96)	0.54 (0.25–0.80)	0.240
**IgM day3 [g/L]**	0.59 (0.47–0.88)	0.40 (0.36–0.62)	0.141
**IgM day5 [g/L]**	0.59 (0.39–1.06)	0.61 (0.32–0.87)	0.606
**κ day1 [g/L]**	2.55 (1.85–3.21)	2.37 (2.02–3.86)	0.844
**κ day3 [g/L]**	2.64 (1.99–3.12)	2.80 (2.06–3.72)	0.615
**κ day5 [g/L]**	2.71 (1.83–3.17)	2.99 (2.33–4.05)	0.274
**λ day1 [g/L]**	1.77 (1.18–2.25)	1.61 (1.18–2.29)	0.486
**λ day3 [g/L]**	1.61 (1.15–2.32)	1.63 (1.17–2.53)	0.541
**λ day5 [g/L]**	1.56 (1.24–2.39)	1.73 (1.33–3.00)	0.530
**κ/λ day1**	1.58 (1.26–1.86)	1.75 (1.38–1.92)	0.219
**κ/λ day3**	1.64 (1.31–1.75)	1.71 (1.43–1.91)	0.734
**κ/λ day5**	1.60 (1.34–1.73)	1.71 (1.41–1.79)	0.456

WBC, white blood cell count; NEUT, neutrophil count; NEUT%, neutrophil ratio; LYMPH, lymphocyte count; LYMPH%, lymphocyte ratio; MONO, monocyte count; MONO%, monocyte ratio; CRP, C-reactive protein; C3, complement C3; C4, complement C4; IgA, immunoglobulin A; IgG, immunoglobulin G; IgM, immunoglobulin M; κ, immunoglobulin κ chain; λ, immunoglobulin λ chain.

Parameter reported as median (IQR).

a Mann–Whitney U test comparing 28-day survivors with nonsurvivors.

### Differential transcriptional signature for SAD

To further explore the potential factors that were associated with SAD and 28-day mortality, we profiled the transcriptome of PBMCs isolated from four surviving patients (i.e., the survival group) with sepsis but not delirium and three nonsurviving patients (i.e., the nonsurvival group) with both sepsis and delirium on days 1, 3, and 5. Principal component analysis showed that the nonsurvival group was clustered and separated from the survival group (**Figure 2A**). There were a total of 2,245 DE genes between the two groups (**Figures 2A–C**, **Table S3**). GO and KEGG enrichment analysis showed that these DE genes were mainly associated with immune and metabolism pathways (**Figure 2D**; **Tables S5**, **6**). Furthermore, we grouped these DE genes into nine clusters with k-means clustering (**Figure 2E**). Genes in the same cluster behaved in similar expression patterns as time went by. Genes in cluster 7 had higher expression in the nonsurvival patients, whereas genes in cluster 3 were highly expressed in the survival patients, especially on day 5. KEGG analysis revealed that the DE genes in cluster 3 were associated with Fc gamma R-mediated phagocytosis and metabolism, those in cluster 7 were mainly involved in cytokine–cytokine receptor interaction, and genes of cluster 8 were enriched in several metabolic pathways (**Figure 2F**; **Tables S6**, **7**).

**Figure 3:**
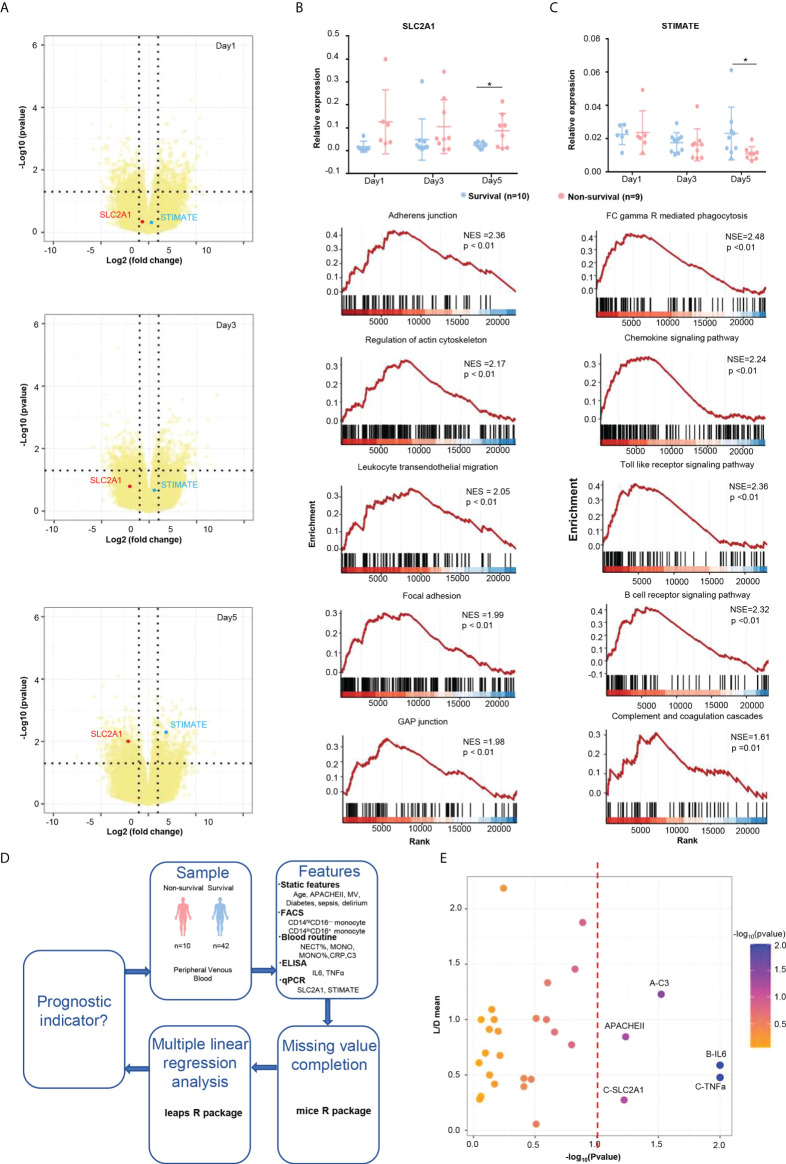
Poor prognostic indicators of ICU patients. **(A)** Volcano plot of the DE genes at days 1, 3, and 5. SLC2A1 is labeled as the red plot, and TIMATE is labeled as the blue plot. **(B)** qPCR validation and GSEA enrichment plots of SLC2A1. The 28-day survivors (blue dots) and nonsurvivors (red dots) were compared by *t*-test. Plots show mean and SD. **(C)** qPCR validation and GSEA enrichment plots of STIMATE. **(D)** Flowchart of prognostic feature analysis. The 28-day survivors (blue dots) and nonsurvivors (red dots) were compared by *t*-test. Plots show mean and SD. **(E)** Linear regression analysis of features that were potentially associated with poor prognosis. * refers to p<0.05.

### GSEA revealed biological function of potential genes associated with ICU mortality

To discover potential biomarker genes to predict the deterioration of the condition, all DE genes were analyzed with the expression patterns obtained by k-means analysis. We focus on clusters that increased (cluster 3) or decreased (cluster 7) over time in the k-means analysis. On the conditions of baseMean > 50, *p*-value <.01, and |log_2_FoldChange|> 1.5, several potential genes were screened and then validated by qPCR. Finally, transcriptome analyses identified two potential genes that could predict the deterioration of the condition (**Figure 3A**) that were experimentally validated by qPCR (**Figures 3B, C**). SLC2A1, a gene from cluster 7, was highly expressed in the nonsurvival group at day 5 (**Figures 2E**, **Figure 3A**). SLC2A1 encodes an important glucose transporter in the mammalian brain blood barrier (BBB), which ensures the energy-independent facilitative transport of glucose into the brain, and its impairment can cause neurological syndrome (14, 15). The metabolism and nerve disease–related KEGG pathways were enriched by GSEA analysis of SLC2A1 (**Figure 3B**, **Table S8**), suggesting its role in the metabolic disturbance of the brain and the neurological disorder in the nonsurvival patients. Conversely, STIMATE, a gene from cluster 3, was highly expressed in the survival group at day 5 (**Figures 2E**, **Figure 3A**). STIMATE acts as a regulator of the store-operated Ca2^+^ entry at junctional sites that connect the endoplasmic reticulum and the plasma membrane (16). STIMATE was related to a series of immune-related pathways, suggesting that a good immune status may help to improve the survival quality of ICU patients (**Figure 3C**, **Table S8**).

### Predictive modeling of ICU mortality

Last, to identify key prognostic factors for death in patients admitted to the ICU, we further analyzed the static and dynamic features from patients (**Figure 3D**). Multiple linear regression analysis showed that the high expression of IL-6 and TNF-α and low levels of C3 significantly increased the likelihood of mortality in the ICU, whereas other variables failed to achieve significance in multivariate testing (**Figure 3E**, **Table S9**). Among the variables that were not significant, higher APACHEII scores (*p* = .058) and higher expression of SLC2A1 (*p* = .068) were also worthy of attention (**Figure 3E**, **Table S9**).

## Discussion

In this study, we enrolled a cohort that included 52 patients to identify the risk factors of SAD and mortality in the ICU and determine the underlying molecular mechanisms. The result showed that several immunological features, including CD14^hi^CD16^-^ monocyte prevalence, cytokine concentration, and longitudinal expression of SLC2A1 and STIMATE in peripheral blood, can be potentially used as predictive factors for SAD and patient death in the ICU.

Early and rapid identification of ICU patients with poor prognosis is a great challenge. The effective prognostic indicators are conducive to identifying critically ill patients for intensive care physicians. To identify the risk factors of mortality in the ICU, we recruited a cohort of patients in the ICU to explore the potential prognostic factors. In our cohort of ICU patients, we observed that 19.23% of the admissions died during hospitalization or the follow-up investigation. A high frequency of delirium was observed in nonsurviving patients, especially in sepsis patients, suggesting that SAD is an important risk factor for death in the ICU. The APACHE II scores, age, and ICU stay in days of patients with SAD were significantly higher than those of septic patients without delirium, which is consistent with previous studies (17–19). The 28-day mortality rate in patients who had SAD was 47.1%. It is also consistent with other studies that delirium and SAD are associated with increased mortality in the ICU (20, 21).

There is still much to learn about the pathophysiology of SAD. Microglial and astrocytic activation occur, resulting in neuroinflammation and an increase in the central nervous system (CNS) cytokine levels, thus causing delirium and sickness behaviors (22, 23), and this is one of the major components of the neuroinflammation mechanism in SAD. It is shown that, when microglia are inhibited in rats, their cognitive function is preserved following a septic episode, suggesting that microglial overactivation may play a crucial role in the development of SAD (24). A recent study reported that microglial activation and inflammation in the hippocampal region were consistent in both nonsepsis and septic delirium mice, followed by impaired hippocampal long-term potentiation (25). There is an absence of a direct infection in CNS during SAD (26), so it is still unclear how microglial cells are activated. Systemic inflammation induced by sepsis is accompanied by an increase in pro-inflammatory cytokines as well as oxidative damage to the BBB. With the subsequent influx of pro-inflammatory cytokines into the CNS, microglial cells are activated (24, 27). In addition, BBB leakage results in the infiltration of peripheral monocytes in the brain that participate in the pathogenesis of CNS diseases (28, 29). A study examined the association between peripheral inflammatory cells and sepsis-associated encephalopathy in a mouse model of delirium and revealed that neutrophils and CCR2^+^ inflammatory monocytes were recruited into the brains, and the prevention of CCR2^+^ inflammatory monocyte recruitment could reduce microglial activation and other neuroinflammation (30). In our study, we found that peripheral CD14^hi^CD16^—^ monocytes were significantly lower in patients with SAD. Whether the peripheral CD14^hi^CD16^—^ monocytes can infiltrate and be activated in CNS still needs to be studied further. The detailed mechanism of how peripheral inflammation affects the CNS also remains to be explored.

High mortality in the ICU and the lack of efficient therapies for SAD provide a strong incentive to identify new disease pathways and more reliable biomarkers, notably those that can be used for diagnosis in early stages. Biomarkers for early detection and patient stratification will be even more important in the future when additional therapies for sepsis patients become available. For many years, several potential biomarkers of SAD in different levels have been found (31–33). The role of the proportion and number of peripheral immune cells in the prediction of SAD is one of the research directions. Dongkai Li et al. reported that lymphocyte and NK cell counts were significantly higher in senior patients with SAD, and NK cell count may be valuable for the prediction of SAD within elderly patient cohorts (34). In our study, we demonstrated that CD14^hi^CD16^—^ monocytes were significantly downregulated in patients with SAD. Thus, the proportion of CD14^hi^CD16^—^ monocytes in peripheral blood may be a predictor of SAD. However, the predictive effect of a single biomarker is not accurate due to the complexity of the disease.

Gene expression profiles from ICU patients can provide comprehensive data that can elucidate pathogenesis and host immune responses. It has been applied in the research of sepsis to help people better understand the pathogenesis of sepsis. Zhenhua Li et al. used the least absolute shrink and selection operator (LASSO) regression and found that SLC2A6, C1ORF55, DUSP5, and RHOB might have important implications for the early diagnosis of sepsis patients (35). Kyung Soo Kim et al. used network and gene set enrichment analysis to elucidate sepsis pathogenesis (36). Currently, total transcriptome data of SAD are lacking. In our study, we profiled the transcriptome of PBMCs isolated from four surviving patients (sepsis, nondelirium) and three nonsurviving patients (sepsis, delirium). We found that mortality and SAD-related DE genes were mainly associated with DNA replication, mitochondrial translation, and metabolic processes. We then tested samples from critical patients stratified according to the expression levels of SLC2A1 and STIMATE because their expression patterns were linked to poor outcomes, which could be potential biomarkers to assess the survival status of patients in the ICU. With the development of PCR technology, a transcriptomic molecular tool will be more often applied in the clinic, which can be helpful to evaluate the immune state from peripheral blood, make diagnoses, and stratify patients. Using gene expression profiling to stratify patients is a rapid diagnostic tool, but the available platforms of immune profiling are still immature due to its complexity (37, 38). Beforehand, the panel of transcriptomic markers that can indicate the state of diseases and immune functions still needs to be selected with more validation.

The immune phenotype in critical patients is complex and heterogeneous, so it is better to use multiple biomarkers to evaluate the risk factors of the immune system, which can stratify patients and guide treatment (38). The features with moderate influence (*P* <.1) on mortality were included in the analysis as determined by univariate analysis. Finally, we used multiple linear regression analysis to identify the key prognostic factors for death in patients admitted to the ICU. Our data show that the higher the APACHEII scores, the higher the expressed levels of IL6, TNF-α, and SLC2A1. Additionally, the lower level of C3 significantly increased the likelihood of mortality in the ICU.

Our study proposes multiple markers for stratifying the risk of critical patients and to guide the treatment. There are some limitations to our study. First, this is a pilot study in a single facility with a relatively small group of patients. Second, our results are limited to critically ill ICU patients. Third, the fact that delirium was diagnosed by using CAM-ICU limits the generalizability. Finally, in our patients, delirium was diagnosed without the participation of consulting psychiatrists, which may affect the accuracy of the diagnostic results. Our observations need follow-up studies with larger patient groups for validation and further efforts to reveal the significance of these predictors in future studies.

## Conclusions

In this cohort study, nonsurviving critical patients have a different immune profile. The CD14^hi^CD16^-^ monocyte percentage may be predictors of SAD in ICU patients, and APACHEII scores, IL6, TNFα, SLC2A1, and C3 associated with the likelihood of mortality 28 days after ICU admission. Our study proposes multiple markers for stratifying the risk of critical patients and to guide the treatment.

## Data availability statement

The data presented in the study are deposited in the GEO data repository, accession number PRJNA767452.

## Ethics statement

The studies involving human participants were reviewed and approved by the Ethics Committee at the First Affiliated Hospital of Jinan University. The patients/participants provided their written informed consent to participate in this study.

## Author contributions

ZW and GC conceived of and designed this study. ZW, XZ, and JS provided clinical samples and helped clinical information collection and analysis. WL was responsible for major experiments. YX, JD, and CX helped in cell sorting and FACS experiments. SS, LG, YC, TM, and YL helped in the experiments. PW helped in data analysis and interpretation. OL, ZR, and GC performed data analysis. WL, GC, and ZW wrote the manuscript. All authors contributed to the article and approved the submitted version.

## Funding

This work was supported by grants from the National Key Research and Development Program of China (2018YFC2002003 to GC), the Natural Science Foundation of China (U1801285 to GC, 81971301 and 32050410285 to OL), Guangzhou Planned Project of Science and Technology (201904010111 to GC, 202002020039 to OL), Initial Supporting Foundation of Jinan University (GC and OL), and Guangdong Basic and Applied Basic Research Foundation (2020A1515010538 to ZW).

## Conflict of interest

The authors declare that the research was conducted in the absence of any commercial or financial relationships that could be construed as a potential conflict of interest.

## Publisher’s note

All claims expressed in this article are solely those of the authors and do not necessarily represent those of their affiliated organizations, or those of the publisher, the editors and the reviewers. Any product that may be evaluated in this article, or claim that may be made by its manufacturer, is not guaranteed or endorsed by the publisher.
