# Supplementary material for: Immunological risk factors for sepsis-associated delirium and mortality in ICU patients
Source: Front Immunol. 2022 Sep 20;13:940779. doi: 10.3389/fimmu.2022.940779 (PMC9531264; doi:10.3389/fimmu.2022.940779)
Supplement: Supplementary file 1 [file DataSheet_1.pdf]

Table S1 FCSA data of the sepsis patients with or without delirium

Parameter	Non-delirium	Delirium	P-vaule
T cell day1 [%], mean ± SD	21.84 ± 9.47	18.08 ± 10.02	0.351
T cell day3 [%], mean ± SD	26.17 ± 10.86	21.80 ± 12.16	0.363
T cell day5 [%], mean ± SD	22.47 ± 9.87	22.58 ± 11.52	0.981
B cell day1 [%], mean ± SD	11.13 ± 5.35	10.99 ± 5.41	0.949
B cell day3 [%], mean ± SD	11.99 ± 5.20	9.48 ± 3.80	0.167
B cell day5 [%], mean ± SD	15.32 ± 6.34	12.08 ± 4.15	0.136
NK cell day1 [%], mean ± SD	4.14 ± 3.12	3.47 ± 2.71	0.570
NK cell day3 [%], mean ± SD	2.58 ± 1.30	3.00 ± 2.16	0.582
NK cell day5 [%], mean ± SD	2.74 ± 2.92	2.96 ± 2.56	0.843
$CD14^{hi}CD16^{-}$ day1 [%], mean ± SD	13.99 ± 6.76	6.52 ± 5.92	0.007
$CD14^{hi}CD16^{-}$ day3 [%], mean ± SD	12.91 ± 9.34	7.22 ± 5.50	0.061
$CD14^{hi}CD16^{-}$ day5 [%], mean ± SD	10.73 ± 7.91	7.95 ± 6.39	0.342
$CD14^{lo}CD16^{+}$ day1 [%], mean ± SD	8.54 ± 8.52	9.28 ± 10.29	0.852
$CD14^{lo}CD16^{+}$ day3 [%], mean ± SD	10.10 ± 7.16	7.58 ± 5.18	0.309
$CD14^{lo}CD16^{+}$ day5 [%], mean ± SD	6.25 ± 4.61	9.25 ± 7.11	0.252
$CD14^{hi}CD16^{+}$ day1 [%], mean ± SD	5.94 ± 6.80	4.14 ± 5.25	0.456
$CD14^{hi}CD16^{+}$ day3 [%], mean ± SD	5.94 ± 5.85	5.46 ± 7.08	0.857
$CD14^{hi}CD16^{+}$ day5 [%], mean ± SD	3.35 ± 3.24	4.49 ± 4.45	0.497

Table S2 FCSA and ELISA data of non-survivors and survivors

Examination	Parameter	Survival	Non-survival	P-vaule
FCSA	T cell day1 [%], mean ± SD	22.20 ± 12.51	22.15 ± 10.72	0.992
	T cell day3 [%], mean ± SD	28.06 ± 13.32	20.23 ± 13.01	0.122
	T cell day5 [%], mean ± SD	26.45 ± 13.36	21.65 ± 9.93	0.346
	B cell day1 [%], mean ± SD	11.18 ± 5.22	10.06 ± 4.41	0.557
	B cell day3 [%], mean ± SD	11.26 ± 5.28	8.08 ± 2.94	0.091
	B cell day5 [%], mean ± SD	12.95 ± 5.03	12.30 ± 3.94	0.736
	NK cell day1 [%], mean ± SD	3.73 ± 3.95	3.50 ± 2.43	0.868
	NK cell day3 [%], mean ± SD	2.83 ± 1.97	2.46 ± 2.18	0.625
	NK cell day5 [%], mean ± SD	2.69 ± 2.40	3.47 ± 3.20	0.437
	$CD14^{hi}CD16^{-}$ day1 [%], mean ± SD	9.02 ± 6.31	9.58 ± 9.16	0.831
	$CD14^{hi}CD16^{-}$ day3 [%], mean ± SD	8.08 ± 6.21	8.56 ± 8.74	0.852
	$CD14^{hi}CD16^{-}$ day5 [%], mean ± SD	8.27 ± 6.36	8.00 ± 5.69	0.912
	$CD14^{lo}CD16^{+}$ day1 [%], mean ± SD	5.90 ± 7.54	11.50 ± 9.42	0.066
	$CD14^{lo}CD16^{+}$ day3 [%], mean ± SD	6.20 ± 4.61	10.08 ± 7.15	0.050
	$CD14^{lo}CD16^{+}$ day5 [%], mean ± SD	4.94 ± 4.02	11.99 ± 8.24	0.047
	$CD14^{hi}CD16^{+}$ day1 [%], mean ± SD	3.79 ± 5.38	3.75 ± 1.97	0.984
	$CD14^{hi}CD16^{+}$ day3 [%], mean ± SD	4.43 ± 5.91	3.16 ± 2.72	0.536
	$CD14^{hi}CD16^{+}$ day5 [%], mean ± SD	2.85 ± 3.36	3.76 ± 3.08	0.485
ELISA	IL6 day1 [pg/mL], median (IQR)	14.66 (5.53-64.57)	43.77 (21.41-98.45)	0.078
	IL6 day3 [pg/mL], median (IQR)	9.84 (5.61-22.50)	32.74 (18.70-71.82)	0.014
	IL6 day5 [pg/mL], median (IQR)	7.87 (3.80-12.68)	37.21 (18.66-71.82)	0.001
	IL10 day1 [pg/mL], median (IQR)	10.99 (7.12-24.12)	9.78 (9.56-12.92)	0.915
	IL10 day3 [pg/mL], median (IQR)	8.94 (7.22-13.83)	10.55 (9.78-15.23)	0.067
	IL10 day5 [pg/mL], median (IQR)	8.75 (7.26-14.60)	12.28 (9.50-20.33)	0.102
	TNFα day1 [pg/mL], median (IQR)	10.54 (9.21-12.51)	13.90 (12.04-16.28)	0.054
	TNFα day3 [pg/mL], median (IQR)	11.74 (9.95-15.57)	12.72 (9.13-14.51)	1.000
	TNFα day5 [pg/mL], median (IQR)	10.44 (9.07-12.35)	14.95 (12.65-16.79)	0.007

Table S3 DE Genes at day1, day3 and day5

Day1		Day3		Day5	
**UP-regulation**	**Down-regulation**	**UP-regulation**	**Down-regulation**	**UP-regulation**	**Down-regulation**
P2RY14	HRH4	CSF3	MSC-AS1	WDPCP	ACTBP8
ZNF415	ARL17B	PTGES	AL121652.1	MYO1A	SLC12A8
APOL1	MTHFD1L	MELTF	OR52K1	GSTM4	HIST1H1D
AC018521.5	CAPN11	MFSD2A	MYOM2	AC138969.1	DARS-AS1
PLAGL1	NSFP1	HIC1	DTWD2	MTERF1	PI3
HLA-DRB1	MYOM2	AL663070.1	AC048380.2	ZMAT1	HIST1H1E
AC007731.2	TSPAN12	ANKRD20A4	AL589655.1	COL9A2	AL356273.3
AC215522.3	AL031846.1	HLA-DRB5	IFIT1	SDK1	IFITM10
AC107884.1	EMILIN1	LINC01451	AIM2	AC117383.1	CHPT1
CARD17	LAPTM4B	GPR35	AC007224.2	ULK4P2	ABCA2
AL157871.3	NDFIP2	AL357874.2	MT-TV	PAXBP1-AS1	LIPH
AC006449.3	LYPD3	AC084125.2	AC018450.1	MCCC1-AS1	AL450322.1
AL121985.1	AL353597.3	LRP5L	CHST2	MFGE8	HIST1H2APS3
PLCL1	EPX	BMF	RHBDL2	KCNAB3	LYPD3
AC132942.1	FADS2	RPL35AP32	GTF2E1	SCN5A	TAS1R3
LYRM9	PEG3	TCEAL3	Z98200.1	AC006254.1	AL121757.1
AC025048.2	IGLV2-18	ASMT	AL450322.1	AC012254.3	EVA1B
HRK	STARD13-AS	SLC7A5P1	AC138150.1	CD163	CHST2
GBP3	PHLDB1	AC093525.4	MT-TS2	AC068050.1	RPS29P14
AC138866.2	EBP	MIR3175	AL031777.3	ATP6V1C2	GREB1
STN1	NRSN2	DNMBP	JAKMIP1	VRK2	AC008115.3
ZNF763	PLCD3	ZNF219	LINC02150	CCDC171	MIR663AHG
NEK11	SLC47A1	AL031705.1	AL390066.1	PXYLP1	KCND3
ENPP2	STARD4	AC073487.1	IGKV3D-15	PIAS2	C1orf61
SLFN5	IGLV3-21	THRA	PRRG2	DRC3	SHLD2P2
EBI3	FADS1	HLA-DRB1	FGFR4	RNU1-22P	MYOM2
IL18BP	IGKV2-24	AL121944.1	EPB41	DAAM2	AC073534.1
IL2RA	TEX30	TMEM189	PLGLB1	NNAT	IGKV1D-16
HLA-DRB5	STX1A	SFR1	LPIN2	BORCS7	SHE
AC091045.1	AL137003.1	OPA1-AS1	SMTNL1	ZNF585A	AC004987.2
ALMS1P1	RPS20P22	LINC02285	CYP27A1	AC063950.1	DUSP14
IGFALS	IGHG3	SNHG7	SIAH2	ITIH3	RAMP1
HAPLN3	UBE2SP1	AC092127.2	KAZN	SLC5A10	AC013451.2
CYP4F25P	PSAT1	BCL6B	AC022784.5	PRL	AC022517.1
AL162457.1	AC083880.1	AC010761.3	AL023806.3	GOLGA8K	LY6G6C
AC124319.1	AC073127.1	NRP1	AC005798.1	UBE2V1	NLRP7
SH3BGRL	NET1	AC026401.2	MROH5	POU5F1B	AC012363.2
SP140	ADGRD2	AL031733.2	AC126474.2	AC244093.5	ANP32C
CYP4F60P	NR5A1	ZBTB33	RNA5SP324	AC000120.1	PTMS
ZSCAN16	AC012676.1	AC093627.3	LINC01395	AL450992.3	FAM43A
AL691449.1	CPSF1P1	REPIN1	RNU7-123P	STIMATE	ZDHHC2
CD40	HS3ST3A1	PITPNM3	HIST1H4C	HIC2	AC017083.1
CLCN4	F2R	MIR6774	AC096887.2	AL031985.1	AL035661.1
SLAMF8	KPNA2	AL137792.1	LEP	NAV2	TRBV6-2
CBFA2T3	SLC16A6P1	RPS26P21	ANKRD55	RNA5SP494	AC068831.1
CALHM6	PHKA1	GSTM4	TRBV6-2	KCNIP4	AL162274.2
AL662844.3	HPDL	AC091181.1	OASL	CYP2B7P	AC104695.2
TRIM34	MIAT	CYSRT1	AC109449.1	AC091132.5	AC117382.2
RF00443	AP002761.3	FBN1	FBXL13	PRKCE	CHRND
PARP11	SGO1	AL513497.1	HIST1H4K	AL590999.1	GCSAML
AC009961.3	CISD1	ITIH1	TRPA1	ANKRD20A7P	LINC02193
AC004584.1	AC105760.2	FZD4	AP006621.2	MED9	CSDC2
RNF227	TPM2	AC093535.1	AL591135.1	Z94721.2	KAZN
CYCSP34	SREBF2	LEKR1	ZFX-AS1	AC104452.1	AP003390.1
AC092384.2	AC118465.1	SFTPD	PPP1R2P1	HEYL	AC215522.1
AC017104.1	UNQ6494	IGHV5-10-1	TMEM272	KRT18P7	LINC-PINT
TBC1D32	IGLC3	FTOP1	LINC02416	ACTG1P10	TRAPPC5
AC113404.1	HLTF	RN7SL738P	IGLV3-21	GLYCTK-AS1	SGCD
SRCIN1	NPW	AL449106.1	NPIPB2	PMS2P10	AL450322.2
RMI2	TMEM176A	ETV5	DRAXIN	AC009093.3	AC104695.3
LY6E-DT	IGHG1	AL451074.4	PYY2	MTND5P11	CASQ1
AMACR	GLDC	RRN3P2	SPATA9	CASC2	CXCL5
LINC00189	CICP4	EREG	AL159972.1	AL592158.1	FBXL13
CHMP4C	HIST1H4J	SDK1	AC087741.3	ZBTB16	LSMEM2
SLAMF7	PKD1P1	AL590632.1	CALB1	FAM217A	HIST1H4E

IL15RA	AL513497.1	AC103810.5	LINC02520	RUFY4	RF00019
FTOP1	SNORD69	AC011481.2	HIST1H2BG	EPCAM	ARG2
EPHB6	FGF17	CTNNB1	LINC01232	ABCC11	MAGI3
AC092384.3	NBL1	AL669942.1	AC145212.1	AC027796.5	F8A3
Z97192.1	FOXC1	SNORA3B	AC145138.1	TIMP4	GLI1
ZNF300P1	CCDC3	AC138956.1	BTBD8	AC090673.1	AL353135.1
AC090543.1	RGPD5	AC062017.1	AC007431.3	AL355922.1	NFE4
PDIA3P2	STON1	AL031708.1	AC100763.1	ITIH4	AL513329.1
KLF2	SERPINB2	IGHV1-2	MINPP1	AC063948.1	QPCT
DES	AC005326.1	GRASP	INHBB	AC108449.2	RPL7P50
C3orf14	SNRNP25	DOC2A	FAT1	TMEM110-MUSTN1	LTB
ZSCAN18	PON2	AC116913.1	CFAP77	AC124283.4	ALS2CR12
ZNF609	SNORA2A	TAS2R46	TRAPPC12-AS1	AL390208.1	IL17REL
BAIAP2L2	HMGN1P7	AC105339.5	LPAR1	TMEM198B	EXOC3L4
IKBKGP1	CD9	AC005586.1	AL034417.4	GOLGA8M	AC243830.1
AC073349.1	HMGB3	AC109460.1	NKAPL	AL512506.1	NR1I3
AC007342.5	DIXDC1	SEPT7-AS1	HEXIM1	LYRM9	REEP2
SLC46A1	MIR4668	RN7SL75P	STK11	AC004982.2	FAM89B
LINC02422	VLDLR	AC092127.1	GPLD1	CAPN3	PLEKHF1
AC124319.4	C4orf46	AC026150.1	OR52K2	AC048341.2	RYR2
CD1C	PYY2	NCR3LG1	LINC00381	AC107884.1	RNF5P1
GBP2	IGLV1-44	AC020612.3	RF00494	AF127936.1	C8orf88
TAP1	NACA3P	NRBP2	AL161443.1	AC073592.7	AP001412.1
MYOF	MANF	CR769776.2	RNF123	TMOD2	C12orf75
PSMB9	AC090950.1	AC004253.1	HTR6	SNORA7B	LINC01973
ADGRG2	KAZN	CLCF1	MKKS	SNX18P9	INSRR
AC105265.1	LINC00997	GFRA2	TIMM10	MIR7152	DUSP8
INTS4P1	CKS2	AL512306.1	AL354732.1	MIS18BP1	TNPO1P1
RTEL1-TNFRSF6B	LRRC37A	AC107982.2	SLC6A11	PPFIA4	AL353719.1
LRRC74A	TWSG1	CIR1P2	ZFPM2	DET1	RAPSN
NRXN1	AC007922.1	AC069234.4	NFE4	AP000786.1	AC243829.2
AFMID	FGFR1	AC108879.1	IGLV4-69	AP001432.1	AC243829.1
AC007220.1	LDLR	CCDC173	HEY1	CACNA2D3	GYPC
AC012645.1	RGPD4	ZNF713	BANF1P1	WFIKKN2	SLC22A1
HSBP1L1	ARG2	NRIP1	RPH3A	GLUD1P3	AL035693.1
AC099063.4	IGHG2	NPM1P19	AL031666.1	FRRS1	KLKB1
CACFD1	AC012603.1	MAATS1	LMNB1	PTPRVP	INHBE
MIR4645	NUFIP1	ADSSL1	DDX58	NRBP2	CRAT37
LY75	PLEKHA5	C3orf14	PSPN	ELOCP2	COL11A2
C2	SFXN1	Z94721.3	NHLH1	AL022322.1	S100A4
TRBV6-1	AC008429.2	ZNF629	ATL1	AL590128.1	RNU2-6P
CYB5R2	IGHG4	AC009088.1	ACOXL	AC026150.1	SMIM23
CLIC4P1	TRPC6	KLHDC9	MT-TH	LINC02256	AC245884.2
AC005476.2	AL591926.6	PPIHP1	PCDHGA5	PMS2P4	AC136475.2
CASP5	MAP7D2	RPS6KA2	AC018868.2	AF127577.2	C19orf67
GTF2IP14	HYOU1	AC073592.3	LINC02033	PIMREG	NGEF
SLCO2B1	SMIM1	ANKRD42	GALM	AC024560.3	VCPKMT
CXCL9	FAM96AP2	SFRP1	RPL23AP66	AC005480.1	FAM131B
TMEM229B	AVPR1A	CAVIN4	TRAV1-2	MYO1B	DOK4
ZMAT1	AC112721.1	IFFO2	RNU1-134P	AC243964.3	SLC2A1
AC090607.1	AL133467.1	AC007431.1	MTCO1P28	TTLL1	BEST2
LIX1-AS1	AC106795.2	RPL15P14	ERMAP	AC116021.1	AC012368.2
SEC14L1P1	CREB3L1	FBXW4P1	GALNT8	RAD9B	CAV2
SIRT4	NPM3	RPS26P47	INHBE	ANKRD20A1	PDE2A
MPND	HMGA1	CTTNBP2	SCARF1	CEMIP	HGD
AC008121.2	TTLL7	FRMD6	MT-TM	AC110285.2	AC004921.1
CROT	GCAT	AC005225.4	AL031963.2	ZFY-AS1	AC022217.1
CD1A	HUS1B	C4orf47	RHD	EFCAB14-AS1	HIST1H3A
OCM	TRIB3	HLA-DQB2	MT-TD	ZBTB33	CYP21A1P
TCL1A	AC068831.7	MXRA8	BNIP3L	AC026150.3	ASS1
C19orf12	UBE2C	CIPC	XKR3	ZNF595	COX6B1P5
NDUFA3P2	WFDC1	AL031710.1	RPL23AP40	LGALS9DP	FASLG
ZNF570	NLRP2	CCDC187	SLC6A12	IGLV1-36	GASAL1
LINC02555	IGHV6-1	SRCIN1	RNU6-260P	LNPK	ITGB4
ZNF322	AC135983.4	RPL17P40	RNU6-299P	APCDD1	CCIN
CEACAM5	RAB6B	RNF227	UVRAG-DT	GOLGA8Q	FAM209B
AC131011.1	GREB1L	PRLR	SPSB1	FAM205A	AL365295.1
WDR35	DHCR7	AC106886.3	CARD17	TCN2	SNORD72

CXCL10	PRKCA	GRIFIN	SLC9A4	PACRGL	AC010331.1
PALLD	IGLV2-23	ZNF667	AC067747.1	LINC01579	IL6
GAPDHP14	DHCR24	AC016394.1	AP005131.2	CHRFAM7A	GADD45G
CFAP99	FAM183DP	ZNF618	ZNF890P	AC009119.2	AL031432.3
RBFADN	SPAG5	AC105020.5	FAP	GRIK1	AL133485.2
C10orf111	AC009133.2	AL356057.1	PRDX2	AC012618.2	LHFPL4
NUTM2A	STAC	AP001625.2	AP000779.1	GSTM2	MOXD2P
RNA5SP39	MGP	AC087276.1	MT-TR	RNU4-25P	ZNF250
COL27A1	TIE1	PLPP3	LARP1P1	ALOX15B	SLA2
SIGLEC7	ATP1A4	AC020728.1	VSTM5	PTPN3	NRARP
TRAJ24	SMTN	MIR4673	ELOCP2	DNMBP-AS1	CICP4
ZNF585A	AC006042.3	FBXO36	TMEM86B	PEX7	AD000864.1
MYBPH	RBBP4P1	LINC01252	RN7SL333P	CD101	AL121899.1
SRBD1	SKA1	AC005785.2	NR2E1	LINC00106	HIST2H2AC
RPL27P12	AC005229.4	IRF5	C9orf153	SHPRH	AC011726.1
C5orf56	HMGN1P32	RF00019	HIVEP2	NHLRC3	ASS1P1
SP2-AS1	MIR4744	SDS	AC008014.1	TNKS2-AS1	LINC00885
STX17	AC092142.2	ULBP2	AC018442.1	AL158212.5	RWDD4P1
LINC02175	AP001505.1	DCP1A	ITGB4	DUSP8P5	AC100810.1
ZNF835	FKBP1B	ARMC9	AC091769.1	ZNF90	INSL3
RNU7-123P	AC087392.1	PMS2P10	GSPT1	AC134043.1	UVRAG-DT
COX6CP14	TWIST2	SLC25A25	AC026803.1	AP001893.1	SPSB1
AC007292.3	NXN	FO704657.1	RAP1GAP	ZNF717	SNX32
RNU6-866P	AC079140.1	AC068189.1	ZBED8	AC096533.1	RTN2
AL121890.4	MREG	AL021068.2	RNU4-90P	STS	ELFN2
AL035448.1	CD177P1	LYPD8	CHST8	CEACAM21	AP002884.1
MPV17L	CDCA4	GSTM2	HSD11B1	AL162385.2	AC018904.1
RPS4Y2	CASQ1	AC117409.1	AC127164.1	AL139120.1	C2orf88
SPATA32	POLRMTP1	RF00275	CA8	AC079416.1	KLHL35
ISG20	AC010864.1	STMN1P1	PTGER4P2	AC010487.1	ST6GALNAC1
RF00602	MRPS30-DT	KRT18P7	TSHB	PAXBP1	LINC01750
OGFR-AS1	AC073476.3	ZDHHC1	HIST1H1T	AC009093.7	AC024937.1
ZNF347	EXO1	MTCO3P12	TNFSF10	LINC01535	ZNF254
CD300LD	BUB1	PGBD5	CXCR2	MIR4432HG	CERS4
AC246787.2	CMSS1	AC098851.1	B3GNT9	RPS15AP27	GNAS
EBAG9P1	GJB6	PRC1-AS1	HIST2H2BC	FMN1	BX284668.6
ZNF443	RYR2	AL390039.1	RN7SL862P	AC005162.3	AC010538.1
AKAP7	COCH	AC024270.2	GID4	AC012651.1	MRPL53
ZNF501	CPLX1	ZNF577	TBC1D3K	RPAIN	AC015871.5
AC005551.1	AC022272.1	RELL1	IGLV2-18	IGHD4-17	NCR1
POM121B	THCAT158	AL022238.1	NOS2P3	AC011611.5	CEBPB-AS1
PARP3	ID2	OSR2	MIR2355	RPL7P46	PLAU
AL645924.2	CCNE1	RPS27AP10	FYB1	AC016292.2	AL133245.1
MIR4285	PDCD6IPP2	MYBPC2	INSL6	AC117503.4	AC023157.3
SEC24B-AS1	PLXNB1	SPDYE5	AC063977.2	MIR1301	CLDN11
GAS8	ALDH18A1	PKD1P6-NPIPP1	CYP7A1	ACKR4	KRT86
RBPMSLP	RSPH1	ANKRD20A1	YWHAZP2	RNU6-988P	TREML1
NRP1	PHLDA1	RPS23P8	AC005086.4	SNORD59A	RNA5SP197
FCGR2B	CADM1	CLMN	AL136295.4	RPS6KA2	EGR3
AC006449.2	MIR6761	MIR6859-4	FKBPL	ZNF77	AC010240.3
AC022893.1	TOMM20L	IGHD1-1	BPGM	AC020763.3	SWT1
NXPH4	CDCA5	MIR762HG	TBCEL	AC006378.1	AC245052.3
C19orf84	SELP	H2AFZP6	RF00409	AL137013.1	GK-IT1
C14orf28	MYCT1	AL357874.1	C14orf119	RN7SL558P	AL031775.1
SNX15	MIR3680-1	ATP5PDP4	AL139288.1	ASMTL-AS1	HIST1H1T
RNU6-262P	AC006064.3	AC004672.1	AICDA	AC010530.1	AL357078.1
PDCD1LG2	AC005224.3	ICOSLG	MEP1B	AC118344.2	TJP1
SLC2A14	PKN3	RNA5SP437	FNDC5	SNX29P2	AC097638.1
PPFIA4	IGHV3-74	SNHG9	C20orf144	RANBP17	AC134407.1
AP002360.3	AC009087.1	CFAP43	NPIPB8	LINC00467	MYRFL
AC103810.5	AUNIP	TRNP1	NATD1	HLA-DRB5	C1QTNF12
NCBP2-AS1	CCNB1	RAMACL	AC023509.6	AC135983.5	AP001992.1
RNF213	PTX3	RNA5SP310	AL033397.2	ECHDC3	PPIAP45
HMGN2P46	ENC1	GUSBP5	FSTL4	GPR35	CSNK1G2P1
LINC01118	ANKRD28	RNU6-125P	IFI16	IGHV3-64D	NAT8B
FBXO6	LRP8	AC109460.3	HNRNPA1P62	WDR88	AC026803.2
AL118557.1	AL391988.1	IGHV3-64D	AC008555.1	NYNRIN	LINC00658
GPR137B	GMNN	AC134043.1	LLPH-DT	NDN	AL139246.5

ACE	CYP51A1	LHB	AC008115.3	IL18	TRHDE
FFAR3	LINC00315	AC011495.1	FAM185A	HLA-DQB2	HIST2H2BD
AC012645.4	STARD13	PHF5CP	AC010359.1	SEPT7P9	LINC01977
FXYD7	RNU6-107P	AC024270.1	SLC26A8	AP000892.3	GCNA
AL669942.1	APOBEC3B	KHDRBS3	NUDT11	NRIP1	TGFB2
ZNF561-AS1	JARID2-AS1	DACT3	IRF2	AC245884.9	NARF
AF196972.1	AC133961.1	SNORA7B	RILP	AC105074.1	HPSE
GRIK1	AC110373.1	AL132656.2	TMEM45B	SLC25A24P1	OR2B11
ZDHHC11B	MRPL23	HRK	ITPRIPL1	CIPC	FAM104B
FFAR1	11-Sep	AL031714.1	WWTR1	AL162578.1	TTC39A
POU5F1P3	YES1	SNORA55	FHDC1	PFN1P6	GNG4
ZFP28	PTGFR	PKDREJ	AL136376.1	RN7SKP23	RF00019
FAM85B	ANXA8	AL133259.1	ST6GALNAC1	QRSL1P3	SNAI1
AC138028.1	RADIL	AC009093.3	GPS2P1	AC136352.4	SCO2
AC079921.2	IGLV5-45	AC011479.3	LINC01762	RNU7-20P	HIST1H2AE
AC009121.1	ELOCP2	SNRPCP3	PINK1	FMO4	AC067747.1
MTUS2	AC138627.1	RPL24P2	MT-TK	STARD4-AS1	LSMEM1
C20orf197	AL391058.1	LRRN1	ARMH4	SLC17A3	AC018450.1
STAP1	OR2H2	MAPK8IP1	KIF26A	SEMA6A-AS1	GJC2
GAPDHP2	FAM83D	AC002558.1	TOM1L1	RNU6-1262P	ANPEP
CTSC	JCHAIN	SLC16A8	AC015871.4	SRCIN1	AC131159.1
SAMD9L	GLIS2	AC134349.1	RSPH9	AC006539.2	RNU6-1165P
AC092745.1	IRGM	FOSB	TRHDE	C5orf66	NDST3
KCNH8	DCC	AC103810.2	HELB	KCNMA1	RN7SL704P
GRID2IP	RAB11FIP5	SFT2D2	ATP6V0CP4	DPPA4	AL133517.1
NUTM2G	VSTM2L	PDK4	SLC22A1	AC009093.10	GATA2
AP001065.1	NIM1K	RBM24	AGPAT4-IT1	ACTG1P20	MEIS3P2
TRANK1	NPIPP1	C9orf43	CLDN14	RSL24D1P8	AC004264.2
AC012020.1	LAMP5	ZNF503	SLC6A9	CYP4F25P	AMZ2P2
TCEA1P2	DOK6	MIR4648	SLC14A1	TBC1D22A-AS1	RNU6-810P
LINC02014	NCAPH	AC005342.2	AC006001.2	RN7SKP151	AL023653.1
DNAAF4	JAKMIP3	AL645933.2	INSYN2	FAM166B	LGALS9C
PPIAP41	IGLV4-69	AL110118.2	CATSPER3	MOCS1	AC245052.2
BTN2A2	AC087721.1	MIR5010	ZNF341-AS1	SCARNA6	LINC00989
AC020911.2	C18orf65	DLL1	PTP4A3	AL391807.1	AC010608.2
AC069234.1	STRIP2	AL356599.1	OXTR	AC022784.1	FHL2
ERVW-1	AL109918.1	RNA5SP212	ZNF547	KCNH8	SLC25A47
TCN2	MMRN1	AC007598.1	NR5A1	AC090617.2	AC019226.1
AC010615.1	AC106739.1	MIRLET7I	CPOX	AC103858.1	RNF103-CHMP3
RNU6-1100P	EPHB2	RN7SL32P	ATP7B	BOLA2	AL391069.2
ZNF470	LRRC37A2	AL732314.6	RHCE	PTX4	BMP6
CLEC17A	UNC13B	GSTM5	AC127502.3	RF00019	CYP4A22-AS1
SNX18P9	MYBL2	AC117500.2	RN7SL166P	AC007496.1	AC058791.1
CBY1	AC092757.2	GAPDHP14	MNDA	RNU5D-1	NQO2
C3	ITGA3	AL157871.3	TRIM55	HLA-DRB1	PLEKHA6
TGM1	AC099654.2	ANKRD24	TRHDE-AS1	GLYATL1	AC087239.1
SFRP1	RF00017	AL136984.1	AC011092.2	ADGRG6	IPO11
AL512288.2	MSMO1	S1PR2	AL162578.1	OCRL	LINC01703
AC090673.1	MIR4284	AC099804.1	AL121983.2	FLVCR2	PDZD2
HNRNPA3P15	AC092045.1	AC114982.2	CSRP2	SPP1	ALKAL2
SNRPCP4	IGHV7-4-1	AC104365.2	ABCB6	AC138932.5	TRAV25
RETREG1	KCNN3	LINC01637	AC010285.1	MIR6719	AC021016.2
AC018797.1	SNORA54	AC010503.4	ACSL6	SNORA2B	MACO1
RF00019	PNCK	CLIC4	DCC	AC009120.1	AC068580.4
CYP2A7	PHF2P2	LINC01150	AL589843.1	ELOCP31	AP000894.4
RN7SL337P	SAMD14	RN7SL413P	TRMT1L	SYT9	RAB39B
AC012358.2	AC015849.1	AC124312.4	COQ3	AL031770.1	GATA2-AS1
SLC49A3	TCHH	DDTP1	FGF8	AL031600.1	AC018638.7
RN7SL842P	GADD45G	CD248	SDHDP6	Z85996.1	AL158801.3
GPR173	FAM131B	STAG3L1	LINC02506	AC009119.3	SARDH
AC092384.1	CYP27B1	TAS2R31	AC016734.1	AL031710.1	TRBV7-2
AC131235.3	FAM184A	CYP4F25P	YWHAQP5	HNRNPA1P68	AC133435.1
SOBP	MIR106B	ST7-OT4	HIST1H4H	FRMD6-AS1	AC055822.1
RSPH4A	HSP90B1	AL049780.1	AL031123.2	CAPS2	AC004846.2
AC009119.2	RF00026	IFI30	DHRS13	CYP21A2	SLC6A13
OLFM5P	LYZ	TEX22	RAB23	GOLGA8H	ISCA1
AL512430.2	TIMP3	MTND5P1	RAB28P5	RNASE7	HIST2H2AA4
LINC02328	SQLE	LINC01932	GLT8D2	ZNF736	UPB1

CD72	CKAP2L	Z69706.1	AL445363.3	ARHGEF34P	FSTL4
CAPS2	AC093206.1	AL035252.3	AC108673.3	AL590282.2	AL078604.2
AL021396.1	GP1BA	AL137186.1	OLIG2	FAM160A1	AL391832.3
CCDC62	HEY2	RF00017	PPP1R2P6	AL645933.2	AC020934.2
NDUFA3P1	AP002852.1	HLA-L	AL358334.3	AC242988.2	AC006001.2
ITPRIPL2	TRIP13	AC008735.1	DNM1P38	AL513365.2	TBC1D3L
IPO4	SLC38A5	RPL39P36	AC097504.1	NUDT16	KIF28P
ZNF780B	ZNF334	IGHD5-12	RNA5SP290	SEMA6B	HIST2H2BE
PRCD	NUF2	RNU6-638P	AL512504.1	AP001363.2	SMIM22
IRF1	ASRGL1	MTCYBP21	FAM183DP	ZNF579	AC061999.1
LINC01841	TGFB1I1	AC079313.1	UBE2L6	SNORD53	AC091825.1
AK4P4	FEN1	MIR324	OR7E100P	AC233280.2	AC132872.2
AC241377.4	MIR618	RDM1P5	MAP2K4	AL109614.1	SPSB3
EIF5AP3	AC092115.1	FAM81A	LIPH	AC126615.2	NSG2
LCA5L	ANKRD55	RPS3P6	SCRG1	SRP14P2	TMEM236
AC005377.1	AC007906.1	ITCH-AS1	NDUFB2-AS1	PDK4	AC008073.1
APOL2	MT-TG	CDC42EP2	LY6G6C	MATR3	AC007611.1
LINC01146	PRKAR1B	Z97652.1	SMAD9	PIGM	FABP5P7
SMIM11A	SFRP4	GNA14	FASLG	AHCYP2	LIF
PGAM1P6	REEP1	ITPK1-AS1	AL078604.2	AL157902.2	DGCR9
KRT18P8	TRBV5-1	PPIAP37	RPL13AP20	AMPH	AC010507.1
FAM225B	CKB	TAGLN	EIF1AX-AS1	TRPM1	RIPOR3
SLC25A24P1	SHCBP1	ZNF229	HEPACAM2	AL138721.1	BOLA3-AS1
LINC01355	HIST1H4B	RBMS2P1	PTP4A2P2	FPGT-TNNI3K	FAXDC2
AC025171.3	AL592166.1	SPATA20	MTND4P26	SLC22A31	MT-RNR2
C22orf42	BEX1	RN7SKP74	SRSF10P1	AL109947.1	FHDC1
AC009041.1	CDKN3	AL158070.1	AC090877.1	SCARB1	MTMR9LP
HPYR1	AC132872.2	AC145285.1	AVPR1A	AL035448.1	MMD
AC020916.1	IGLV2-11	AL606517.2	MT-TG	HLCS	AC011511.1
TMEM74B	TMEM176B	AP000943.4	MIR133A1HG	AC009093.6	AL137060.1
AC111170.3	AL133499.1	CAPNS2	AL512353.1	INSR	TNNT3
FBLN2	GPRC5D	LMNA	S100A5	RPL18AP7	AC026367.3
ZNF66	EPAS1	AC005776.1	PXDNL	AC074029.3	GRTP1
NIFKP6	RNU6-431P	SLC29A1	FAM92B	AL356320.2	SMARCA5-AS1
BANK1	SLC12A2	AP000892.2	TRIM21	DUXAP7	AC116353.5
AC133644.3	PRG2	RPS2P45	KCNA2	ZNF629	RF00017
AC091180.2	ZFPM2	FOXJ1	HIST1H2BF	USHBP1	AL358781.2
AP001962.1	CEP170P1	RF00212	OR52K3P	AC103810.5	ARHGAP9
COX7CP1	PTTG1	AL049539.1	FAM131B	AC004034.1	AL355390.1
DOCK4-AS1	CAND2	RF00019	AC104653.1	AL031705.1	SNORC
CFB	AL157371.2	AL356805.1	DRP2	AL391058.1	AC008115.1
AC016722.2	TMEM97P1	SLC9A5	LRRC37A2	TAGLN	RNU5A-3P
RHOBTB3	SMC4	AC020916.1	KANK2	CTAGE8	CA8
ESR2	AC113382.1		TNXB	DPRXP1	RNU6ATAC18P
AC073359.1	IGKC		HIST1H4D	SUGT1P4-STRA6LP	AL118516.1
AP000593.3	IGKV1-5		IGHV3-53	AL691447.3	AC073172.1
ABCG1	AL136531.3		RPEP6	AL121950.1	AC136628.3
CPNE4	PRTN3		CBX3P4	MRPS18AP1	LINC00862
CACHD1	SEPT14P12		UROD	AMOTL1	SLC22A13
RCAN2	NDC80		DIXDC1	CFAP97D1	AC097376.1
ACTBP14	IGLC2		AC073912.1	AC010973.2	AC092198.1
ABCB4	KLF15		RN7SKP271	CCT8P1	MIR4296
CORIN	AC004884.1		NLRP7	BTBD18	GDF15
AC107959.1	BOLA2B		AC105749.1	HM13-IT1	CYP2D7
IFI35	PLCXD2-AS1		APOL2	GAS6-AS1	CSF1
MIR6772	WASF3		APOBEC3B-AS1	AL132780.4	MYBPC3
GIMAP6	FOXD1		LINC01287	RPL35P3	TMPRSS9
AC004771.3	AC025287.4		PPP1R27	AC087163.3	STK17A
EPB41L5	DPF1		GADD45G	CYP46A1	TMC5
ADGRB3	CCDC169		AC087627.1	AC005586.1	CCDC27
LRGUK	AC006452.1		CDC42BPA	MFSD1P1	SH2D5
ZNF404			AC105384.1	EFCAB13	AC016831.6
AC021106.2			RANP4	AC018695.4	DLGAP1-AS2
SEPT14P4			OSBP2	LINC01352	ATP7B
EFCAB2			SOCS5P4	SRGAP1	AL020997.3
AC007342.8			PDYN	SOX10	ATP1A4
AP001610.1			SHISA5	DNMBP	TCF7L1
VWA3B			HAVCR1P1	AL355075.5	IGLV3-25

GUSBP4	RMI1	ENPP7P10	SNAI3-AS1
GPR79	KLLN	AL928654.2	SPOCD1
AC019080.3	OR2T8	LINC01732	TMEM54
BET1P1	AC069542.1	GRAMD1B	AL049634.2
IL1B	CISD1	SALL4	OSGEPL1-AS1
NAV2	SCARNA8	UBE2Q2P1	PIP
IGHV5-10-1	ELOVL6	AL139811.1	EIF2AK1
SERPINB9P1	RMDN2-AS1	AC018557.2	TWIST1
RN7SL363P	AC016747.4	CDNF	AC007240.2
AL161756.3	EIF2AK1	HRK	LINC01134
FOSB	GJC2	TMEM220-AS1	AC004233.4
BTN3A1	AC025031.2	AC009065.5	CXCL1
RN7SL395P	RF01210	CCDC40	SCN1B
NPM1P37	CFL1P1	AC027607.1	HIST1H2AB
CRB3	REEP1	AC124944.2	OR52W1
AC243964.2	SERPINI1	AL033527.5	AC117394.2
HM13-IT1	AC084781.1	EFCAB6	AL137159.1
ARHGAP20	DHRS12	GFOD1	TRPM6
JAKMIP1	GLIS2	MIR3939	HIST1H4B
ARNT2	AL355365.1	PHGR1	RPS29P5
TNFRSF9	AC073850.1	PCSK1	AL138920.1
SUMO2P17	TPRKBP2	CES1	TREML4
AC040160.2	FTH1P22	GAPT	TDP2
PXT1	LINC01140	RGS5	CTAGE3P
SDC3	GCHFR	AC012146.1	OMG
SDSL	RF01225	LINC02014	MYLK
GBP6	KRT18P63	PALLD	TSC22D1
AC079316.1		ANOS1	PGC
PPM1J		PPM1J	AC087379.2
AP001767.4		LIX1	GFI1B
AL136531.1		CR769776.2	B4GALT1-AS1
AC093642.2		AC002059.1	NSFP1
STMN3		CYCSP10	SMIM18
ZNF626		MRPL53P1	ITGB5
EBF1		Z74021.1	YTHDF3-AS1
EXTL3-AS1		MIR3150BHG	AC092135.3
AC124312.4		OR2A42	LIMK1
RF00019		ALDH2	PTCH2
AL627230.4		ITPRIPL2	AP005329.1
CD274		CXXC5-AS1	PIK3R3
ZFP3		RNA5SP129	HIST2H2BF
AC005696.3		AC007537.1	LINC02390
AP000845.1		SLC31A2	AC239799.1
AC067852.2		PKD1P5	FLCN
SECTM1		Z98749.1	AL354920.1
LINC00680		AC091516.1	NDFIP2
		RNASE1	GRB14
		SMIM17	AC023509.4
		LINC00624	CABP7
		ANKRD20A4	AC013400.1
		MYRIP	KRT18
		ZFP28	RHOV
		C3orf14	NATD1
		PLEKHS1	TBC1D3D
		TTYH1	NRSN2
		AL008727.1	AC243967.3
		AC245060.2	CDHR2
		AC087222.1	ACTN3
		AC245140.3	OOSP3
		AL137077.1	AC138035.2
		RF00019	AL358334.2
		ASB14	SLC45A3
		AC016582.2	DRAXIN
		C11orf97	C1orf116
		HOXA10-AS	EPB41L4B
		IGLL1	DHX34
		SLC26A5	TMEM272
		CCDC144B	DHRS12

AC005921.3	NECTIN4
PCBP2-OT1	HIST1H2BG
MROH7	TNFSF4
AC048382.2	AC022730.4
KBTBD11	THCAT158
MYBPC1	OR51R1P
MIR93	HIST2H2BC
AL121672.2	FAM185A
	SFN
	AC005899.1
	KIRREL3
	PF4V1
	AF001548.2
	LINC01762
	AC240565.2
	PPT2
	AP1M2
	BAIAP2
	PHF13
	RPL3P7
	AC007950.2
	AP000346.2
	CD9
	RNU6-1136P
	AC008277.2
	HIST1H4H
	CCDC96
	AL031316.1
	AC092620.1
	AL662899.1
	AC092910.3
	RPL7P19
	CDKN1B
	AC008467.1
	GRK5-IT1
	MEIS1
	AC008105.3
	DNAH14
	AL139241.1
	HSD52
	CPS1
	ZNF503-AS2
	GPAT3
	FRMD3
	GZMB
	RF00404
	AL136376.1
	GAD1
	GPR61
	AC239803.3
	CTSW
	SCN2B
	CA3-AS1
	GUK1
	CCDC36
	CDK5RAP2
	AP003031.1
	11-Sep
	ADORA2A-AS1
	AC093673.1
	TIGD4
	EGFL7
	U2AF1L5
	MPP1
	DBN1
	CCT5P1
	AC079907.2
	DOK6

GABARAP
LINC01136
AP004609.3
GPR137C
RPS15AP24
LARP1P1
AL512274.1
ACSL6
AL021807.1
ESAM
AC104561.3
AC026368.1
ABALON
MAP2K3
ALOX12B
PRTFDC1
AL136115.2
CTBP2P8
C10orf55
DNAJC27-AS1
C16orf45
AL137789.2
ATP8A2P2
HBG1
CYP26A1
AC034111.1
LINC01730
RNU6-790P
CNN2P1
CCDC71L
GCNT1P3
PLEKHG6
TRY2P
KDELR3
FAM81B
AC018904.2
MANSC1
TMEM45B
HIST1H1PS1
RAB11FIP5
AC016586.1
COQ3
SELP
AP001000.1
SPARC
AC084026.2
AL133163.2
JAKMIP2
YBX2
NIFKP4
AC063977.2
RPS29P20
AC099489.1
MT-RNR1
PIANP
SLC37A3
MIR643
KRTAP5-AS1
STON2
INHBB
LINC01890
IL10RB
SNPH
AKAP12
AC004895.1
FAM222A
AL132780.1
OR52K3P

RAB27B
CPNE9
CNTD2
RNU6-190P
PAPLN
PPP4R1L
AC105277.1
TRHDE-AS1
TLR10
PELO
CICP23
SGO1-AS1
UNC5A
IPMK
AL355073.1
EGLN3
LINC00514
NOP2
RORB
TRAV17
PPIAP29
CNIH3
RILP
Z82206.1
ISYNA1
FBXO2
LINC00672
AC100835.1
BOLA2B
AC092718.6
AC106739.1
PGF
TMEM240
AL512288.1
AC007192.1
STX1A
AL669818.1
Z99774.1
AL356055.1
OR52M2P
GNG11
CTRC
TRAJ33
PIK3R2
LANCL3
MIAT
AL021707.1
FNDC7
IL6R-AS1
AP000275.1
COL18A1
SOD1P1
COL3A1
PINK1
AC011445.2
NRXN3
AL355073.2
HIST1H4J
ADORA2A
NLRP9P1
KMT2E-AS1
AC020661.1
MSC
PTGES3L
AC008280.3
LBX2
GJB6
AC136475.1

KIR3DL2
AL683807.2
AC103563.2
CCDC13-AS1
RN7SL297P
AC145207.5
AC126283.1
AOC4P
AC104241.2
C7
GJB7
AC093155.2
AC092436.4

Table S4 KEGG pathway for DE genes of non-survival group and survival group

	KEGG ID	Pvalue	OddsRatio	ExpCount	Count	Size	Term
	4640	1.08E-07	13.8963	0.83981	9	88	Hematopoietic cell lineage
	5150	1.16E-05	14.1135	0.52488	6	55	Staphylococcus aureus infection
	4514	0.00024	6.44671	1.26926	7	133	Cell adhesion molecules (CAMs)
	4672	0.00106	10.0839	0.45808	4	48	Intestinal immune network for IgA production
	5322	0.00174	5.24492	1.29789	6	136	Systemic lupus erythematosus
	5310	0.00278	12.1279	0.2863	3	30	Asthma
	5140	0.00473	6.49774	0.68712	4	72	Leishmaniasis
	5330	0.00508	9.61931	0.3531	3	37	Allograft rejection
	5332	0.00679	8.60079	0.39127	3	41	Graft-versus-host disease
	4940	0.00776	8.16792	0.41036	3	43	Type I diabetes mellitus
	2010	0.00827	7.96733	0.4199	3	44	ABC transporters
	4060	0.01234	3.0753	2.52897	7	265	Cytokine-cytokine receptor interaction
	5144	0.01241	6.79717	0.48671	3	51	Malaria
**Day1-Up**	5320	0.01308	6.6573	0.49625	3	52	Autoimmune thyroid disease
	4145	0.01481	3.75199	1.46012	5	153	Phagosome
	4620	0.01584	4.48509	0.97342	4	102	Toll-like receptor signaling pathway
	4977	0.02158	9.74747	0.22904	2	24	Vitamin digestion and absorption
	4610	0.02768	4.92796	0.65849	3	69	Complement and coagulation cascades
	5416	0.02873	4.85356	0.66803	3	70	Viral myocarditis
	4612	0.03546	4.44999	0.72529	3	76	Antigen processing and presentation
	4146	0.0391	4.2721	0.75392	3	79	Peroxisome
	5340	0.04354	6.48597	0.33401	2	35	Primary immunodeficiency
	5143	0.04354	6.48597	0.33401	2	35	African trypanosomiasis
	5323	0.05549	3.68182	0.86844	3	91	Rheumatoid arthritis
	232	0.06495	17.5939	0.0668	1	7	Caffeine metabolism
	5142	0.07638	3.20064	0.9925	3	104	Chagas disease (American trypanosomiasis)
	4623	0.09939	3.94925	0.53442	2	56	Cytosolic DNA-sensing pathway
	100	1.26E-09	52.7279	0.22989	7	19	Steroid biosynthesis
	4020	0.00527	3.62031	2.14162	7	177	Calcium signaling pathway
	260	0.00655	8.77485	0.38718	3	32	Glycine, serine and threonine metabolism
	4114	0.0111	4.0286	1.35515	5	112	Oocyte meiosis
	4110	0.01667	3.61472	1.50034	5	124	Cell cycle
	4970	0.02222	4.01194	1.07686	4	89	Salivary secretion
	5215	0.02222	4.01194	1.07686	4	89	Prostate cancer
	1040	0.02613	8.81465	0.25409	2	21	Biosynthesis of unsaturated fatty acids
	330	0.02712	4.97059	0.65337	3	54	Arginine and proline metabolism
**Day1-Down**	4972	0.03338	3.50823	1.22205	4	101	Pancreatic secretion
	5200	0.04196	2.18788	3.94444	8	326	Pathways in cancer
	4115	0.04866	3.8905	0.82277	3	68	p53 signaling pathway
	5310	0.05051	5.97205	0.36299	2	30	Asthma
	4520	0.0579	3.60945	0.88327	3	73	Adherens junction
	4260	0.06584	3.41196	0.93166	3	77	Cardiac muscle contraction
	750	0.07047	16.5486	0.0726	1	6	Vitamin B6 metabolism
	5410	0.07864	3.15276	1.00426	3	83	Hypertrophic cardiomyopathy (HCM)
	4640	0.09007	2.96471	1.06476	3	88	Hematopoietic cell lineage
	4960	0.09121	4.17174	0.50818	2	42	Aldosterone-regulated sodium reabsorption
	5414	0.09483	2.89554	1.08896	3	90	Dilated cardiomyopathy
	4672	0.00304	11.6909	0.29448	3	48	Intestinal immune network for IgA production
	480	0.00341	11.1896	0.30675	3	50	Glutathione metabolism
	980	0.00911	7.70588	0.43558	3	71	Metabolism of xenobiotics by cytochrome P450
	982	0.00983	7.48312	0.44785	3	73	Drug metabolism - cytochrome P450
	4612	0.01097	7.17186	0.46626	3	76	Antigen processing and presentation
	5310	0.01429	12.1933	0.18405	2	30	Asthma
	4640	0.01629	6.14652	0.53988	3	88	Hematopoietic cell lineage
	5330	0.0213	9.74286	0.22699	2	37	Allograft rejection
	4080	0.02394	3.36173	1.66871	5	272	Neuroactive ligand-receptor interaction
	5332	0.02583	8.73756	0.25153	2	41	Graft-versus-host disease
**Day3-UP**	4940	0.02823	8.30846	0.2638	2	43	Type I diabetes mellitus
	4742	0.04006	6.80235	0.31902	2	52	Taste transduction
	5320	0.04006	6.80235	0.31902	2	52	Autoimmune thyroid disease
	5217	0.04435	6.41398	0.33742	2	55	Basal cell carcinoma
	5150	0.04435	6.41398	0.33742	2	55	Staphylococcus aureus infection
	4514	0.0472	3.98741	0.81595	3	133	Cell adhesion molecules (CAMs)
	4310	0.06329	3.51577	0.92025	3	150	Wnt signaling pathway
	4145	0.06636	3.44364	0.93865	3	153	Phagosome
	5416	0.06815	4.98616	0.42945	2	70	Viral myocarditis
	4630	0.06845	3.39713	0.95092	3	155	Jak-STAT signaling pathway
	5140	0.07159	4.84202	0.44172	2	72	Leishmaniasis
	5412	0.07508	4.70588	0.45399	2	74	Arrhythmogenic right ventricular cardiomyopathy (ARVC)
	120	1.24E-02	13.5621	0.17178	2	16	Primary bile acid biosynthesis
	5160	0.01405	3.7931	1.43865	5	134	Hepatitis C
	4920	0.03607	4.41538	0.73006	3	68	Adipocytokine signaling pathway
	3320	0.03882	4.28209	0.75153	3	70	PPAR signaling pathway
**Day3-Down**	512	0.0407	6.76464	0.32209	2	30	Mucin type O-Glycan biosynthesis
	4350	0.06086	3.53333	0.90184	3	84	TGF-beta signaling pathway
	4060	0.06357	2.25401	2.84509	6	265	Cytokine-cytokine receptor interaction

	4080	0.0703	2.19193	2.92025	6	272	Neuroactive ligand-receptor interaction
	130	0.07281	15.5887	0.07515	1	7	Ubiquinone and other terpenoid-quinone biosynthesis
	860	0.07737	4.60936	0.46166	2	43	Porphyrin and chlorophyll metabolism
**Day5-Up**	982	0.00173	8.78261	0.52249	4	73	Drug metabolism - cytochrome P450
	5323	0.00386	6.94374	0.65133	4	91	Rheumatoid arthritis
	4977	0.01249	13.1909	0.17178	2	24	Vitamin digestion and absorption
	980	0.01391	6.51357	0.50818	3	71	Metabolism of xenobiotics by cytochrome P450
	780	0.01426	142.073	0.01431	1	2	Biotin metabolism
	5310	0.01917	10.3536	0.21472	2	30	Asthma
	4145	0.02307	4.0106	1.09509	4	153	Phagosome
	5330	0.02844	8.27286	0.26483	2	37	Allograft rejection
	5332	0.03439	7.41923	0.29346	2	41	Graft-versus-host disease
	4940	0.03753	7.05488	0.30777	2	43	Type I diabetes mellitus
	4672	0.04585	6.28261	0.34356	2	48	Intestinal immune network for IgA production
	4930	0.04585	6.28261	0.34356	2	48	Type II diabetes mellitus
	480	0.04935	6.01875	0.35787	2	50	Glutathione metabolism
	5320	0.05295	5.776	0.37219	2	52	Autoimmune thyroid disease
	5150	0.05851	5.44623	0.39366	2	55	Staphylococcus aureus infection
	140	0.06041	5.34444	0.40082	2	56	Steroid hormone biosynthesis
	590	0.06623	5.06053	0.42229	2	59	Arachidonic acid metabolism
	4122	0.06936	15.7642	0.07157	1	10	Sulfur relay system
	5416	0.08904	4.23382	0.50102	2	70	Viral myocarditis
	5140	0.09341	4.11143	0.51534	2	72	Leishmaniasis
**Day5-Down**	5146	5.74E-05	5.30312	2.18575	10	106	Amoebiasis
	5211	0.00054	5.53996	1.44342	7	70	Renal cell carcinoma
	4060	0.00102	2.86495	5.46438	14	265	Cytokine-cytokine receptor interaction
	5410	0.00151	4.58183	1.71149	7	83	Hypertrophic cardiomyopathy (HCM)
	5414	0.00242	4.19023	1.85583	7	90	Dilated cardiomyopathy
	5323	0.00258	4.13962	1.87645	7	91	Rheumatoid arthritis
	4520	0.00375	4.4231	1.50528	6	73	Adherens junction
	5412	0.00401	4.35729	1.5259	6	74	Arrhythmogenic right ventricular cardiomyopathy (ARVC)
	4510	0.00796	2.6349	4.12406	10	200	Focal adhesion
	5332	0.00967	5.27605	0.84543	4	41	Graft-versus-host disease
	4960	0.01052	5.1363	0.86605	4	42	Aldosterone-regulated sodium reabsorption
	4973	0.01237	4.87778	0.90729	4	44	Carbohydrate digestion and absorption
	5142	0.01998	3.00745	2.14451	6	104	Chagas disease (American trypanosomiasis)
	5144	0.0204	4.14621	1.05164	4	51	Malaria
	250	0.02746	5.01286	0.65985	3	32	Alanine, aspartate and glutamate metabolism
	4810	0.03115	2.18343	4.39213	9	213	Regulation of actin cytoskeleton
	5200	0.03561	1.90487	6.72222	12	326	Pathways in cancer
	5215	0.03609	2.90589	1.83521	5	89	Prostate cancer
	5210	0.03837	3.35337	1.27846	4	62	Colorectal cancer
	4062	0.04076	2.17709	3.89724	8	189	Chemokine signaling pathway
	5212	0.05572	2.94276	1.44342	4	70	Pancreatic cancer
	4940	0.05812	3.62733	0.88667	3	43	Type I diabetes mellitus
	4976	0.05814	2.89833	1.46404	4	71	Bile secretion
	4620	0.05884	2.51066	2.10327	5	102	Toll-like receptor signaling pathway
	4650	0.06125	2.25431	2.80436	6	136	Natural killer cell mediated cytotoxicity
	5220	0.06316	2.81333	1.50528	4	73	Chronic myeloid leukemia
	410	0.07451	4.81261	0.45365	2	22	beta-Alanine metabolism
	910	0.08054	4.58263	0.47427	2	23	Nitrogen metabolism
	4974	0.08539	2.51748	1.67025	4	81	Protein digestion and absorption
	4670	0.09075	2.18857	2.39196	5	116	Leukocyte transendothelial migration
	4150	0.09131	2.95642	1.07226	3	52	mTOR signaling pathway
	5213	0.09131	2.95642	1.07226	3	52	Endometrial cancer
	4350	0.0946	2.42179	1.73211	4	84	TGF-beta signaling pathway
	4512	0.09776	2.39147	1.75273	4	85	ECM-receptor interaction
	330	0.09957	2.83948	1.1135	3	54	Arginine and proline metabolism
	5110	0.09957	2.83948	1.1135	3	54	Vibrio cholerae infection

Table S5 GO terms (BP) for DE genes of non-survival group and survival group

	GO.ID	Term	Annotated	Significa	Expected	pvalue	FDR	score
	GO:0019883	antigen processing and presentation of e...	24	6	0.24	1.20E-07	6.00E-05	4.22185
	GO:0002819	regulation of adaptive immune response	168	12	1.71	1.60E-07	8.00E-05	4.09691
	GO:0002475	antigen processing and presentation via ...	14	5	0.14	1.90E-07	9.50E-05	4.02228
	GO:0002250	adaptive immune response	701	24	7.13	2.10E-07	0.00011	3.97881
	GO:0002460	adaptive immune response based on somati...	370	17	3.76	2.60E-07	0.00013	3.88606
	GO:0001913	T cell mediated cytotoxicity	44	7	0.45	2.80E-07	0.00014	3.85387
	GO:0002822	regulation of adaptive immune response b...	153	11	1.56	4.90E-07	0.00025	3.61083
	GO:0002824	positive regulation of adaptive immune r...	105	9	1.07	1.30E-06	0.00065	3.18709
	GO:0001914	regulation of T cell mediated cytotoxici...	35	6	0.36	1.30E-06	0.00065	3.18709
	GO:0001906	cell killing	172	11	1.75	1.60E-06	8.00E-04	3.09691
	GO:0001909	leukocyte mediated cytotoxicity	108	9	1.1	1.60E-06	8.00E-04	3.09691
	GO:0002821	positive regulation of adaptive immune r...	110	9	1.12	1.90E-06	0.00095	3.02228
	GO:0002706	regulation of lymphocyte mediated immuni...	154	10	1.57	4.10E-06	0.00205	2.68825
	GO:0051250	negative regulation of lymphocyte activa...	154	10	1.57	4.10E-06	0.00205	2.68825
	GO:0002449	lymphocyte mediated immunity	366	15	3.72	5.50E-06	0.00275	2.56067
	GO:0050776	regulation of immune response	1027	27	10.44	5.90E-06	0.00295	2.53018
	GO:0032943	mononuclear cell proliferation	286	13	2.91	7.80E-06	0.0039	2.40894
	GO:0001916	positive regulation of T cell mediated c...	28	5	0.28	8.40E-06	0.0042	2.37675
	GO:0002709	regulation of T cell mediated immunity	73	7	0.74	9.40E-06	0.0047	2.3279
	GO:0002703	regulation of leukocyte mediated immunit...	211	11	2.15	1.10E-05	0.0055	2.25964
	GO:0002456	T cell mediated immunity	104	8	1.06	1.10E-05	0.0055	2.25964
	GO:0001910	regulation of leukocyte mediated cytotox...	75	7	0.76	1.10E-05	0.0055	2.25964
	GO:0002711	positive regulation of T cell mediated i...	50	6	0.51	1.10E-05	0.0055	2.25964
	GO:0006955	immune response	2306	45	23.45	1.20E-05	0.006	2.22185
**Day1-Up**	GO:0002705	positive regulation of leukocyte mediate...	138	9	1.4	1.20E-05	0.006	2.22185
	GO:0002708	positive regulation of lymphocyte mediat...	106	8	1.08	1.30E-05	0.0065	2.18709
	GO:0001912	positive regulation of leukocyte mediate...	52	6	0.53	1.40E-05	0.007	2.1549
	GO:0002694	regulation of leukocyte activation	605	19	6.15	1.40E-05	0.007	2.1549
	GO:0050670	regulation of lymphocyte proliferation	219	11	2.23	1.60E-05	0.008	2.09691
	GO:0032944	regulation of mononuclear cell prolifera...	221	11	2.25	1.70E-05	0.0085	2.07058
	GO:0002695	negative regulation of leukocyte activat...	184	10	1.87	2.00E-05	0.01	2
	GO:0070661	leukocyte proliferation	313	13	3.18	2.00E-05	0.01	2
	GO:0019884	antigen processing and presentation of e...	185	10	1.88	2.10E-05	0.0105	1.97881
	GO:0002682	regulation of immune system process	1646	35	16.74	2.40E-05	0.012	1.92082
	GO:0002697	regulation of immune effector process	470	16	4.78	2.70E-05	0.0135	1.86967
	GO:0019882	antigen processing and presentation	232	11	2.36	2.70E-05	0.0135	1.86967
	GO:0042098	T cell proliferation	195	10	1.98	3.20E-05	0.016	1.79588
	GO:0046651	lymphocyte proliferation	283	12	2.88	3.50E-05	0.0175	1.75696
	GO:0031343	positive regulation of cell killing	61	6	0.62	3.60E-05	0.018	1.74473
	GO:0070663	regulation of leukocyte proliferation	240	11	2.44	3.70E-05	0.0185	1.73283
	GO:0050865	regulation of cell activation	650	19	6.61	3.80E-05	0.019	1.72125
	GO:0031341	regulation of cell killing	92	7	0.94	4.30E-05	0.0215	1.66756
	GO:0050866	negative regulation of cell activation	207	10	2.1	5.30E-05	0.0265	1.57675
	GO:0042129	regulation of T cell proliferation	167	9	1.7	5.50E-05	0.0275	1.56067
	GO:0050766	positive regulation of phagocytosis	68	6	0.69	6.70E-05	0.0335	1.47496
	GO:0051249	regulation of lymphocyte activation	515	16	5.24	7.90E-05	0.0395	1.4034
	GO:0007520	myoblast fusion	45	5	0.46	9.10E-05	0.0455	1.34199
	GO:0002699	positive regulation of immune effector p...	223	10	2.27	9.90E-05	0.0495	1.30539
	GO:0050798	activated T cell proliferation	46	5	0.47	0.0001	0.05	1.30103
	GO:0002455	humoral immune response mediated by circ...	155	16	2.05	2.50E-10	1.25E-07	6.90309
	GO:0006958	complement activation, classical pathway	141	15	1.86	6.00E-10	3.00E-07	6.52288
	GO:0006956	complement activation	178	15	2.35	1.50E-08	7.50E-06	5.12494
	GO:0030449	regulation of complement activation	115	12	1.52	4.00E-08	2.00E-05	4.69897
	GO:0002433	immune response-regulating cell surface ...	139	13	1.83	4.10E-08	2.05E-05	4.68825
	GO:0038096	Fc-gamma receptor signaling pathway invo...	139	13	1.83	4.10E-08	2.05E-05	4.68825
	GO:0038094	Fc-gamma receptor signaling pathway	142	13	1.87	5.30E-08	2.65E-05	4.57675
	GO:0016064	immunoglobulin mediated immune response	227	16	3	6.30E-08	3.15E-05	4.50169
	GO:0002431	Fc receptor mediated stimulatory signali...	145	13	1.91	6.80E-08	3.40E-05	4.46852
	GO:0019724	B cell mediated immunity	230	16	3.04	7.50E-08	3.75E-05	4.42597
	GO:0042742	defense response to bacterium	348	19	4.59	2.10E-07	0.00011	3.97881
	GO:0002920	regulation of humoral immune response	134	12	1.77	2.20E-07	0.00011	3.95861
	GO:0006910	phagocytosis, recognition	88	10	1.16	2.50E-07	0.00013	3.90309
	GO:0006959	humoral immune response	377	19	4.98	7.10E-07	0.00036	3.44977
	GO:0006909	phagocytosis	382	19	5.04	8.60E-07	0.00043	3.36653
	GO:0000070	mitotic sister chromatid segregation	161	12	2.12	1.60E-06	8.00E-04	3.09691
	GO:0008037	cell recognition	226	14	2.98	2.00E-06	0.001	3
	GO:0000819	sister chromatid segregation	196	13	2.59	2.20E-06	0.0011	2.95861
	GO:0098813	nuclear chromosome segregation	272	15	3.59	3.60E-06	0.0018	2.74473
	GO:0006897	endocytosis	675	25	8.91	3.70E-06	0.00185	2.73283
	GO:0006911	phagocytosis, engulfment	126	10	1.66	6.80E-06	0.0034	2.46852
**Day1-Down**	GO:0002449	lymphocyte mediated immunity	366	17	4.83	8.10E-06	0.00405	2.39254
	GO:0007059	chromosome segregation	334	16	4.41	1.00E-05	0.005	2.30103
	GO:1902652	secondary alcohol metabolic process	162	11	2.14	1.10E-05	0.0055	2.25964
	GO:0050864	regulation of B cell activation	195	12	2.57	1.20E-05	0.006	2.22185
	GO:0051983	regulation of chromosome segregation	107	9	1.41	1.20E-05	0.006	2.22185
	GO:0050853	B cell receptor signaling pathway	135	10	1.78	1.30E-05	0.0065	2.18709
	GO:0099024	plasma membrane invagination	135	10	1.78	1.30E-05	0.0065	2.18709
	GO:0033045	regulation of sister chromatid segregati...	84	8	1.11	1.50E-05	0.0075	2.12494

	GO:0038093	Fc receptor signaling pathway	241	13	3.18	2.00E-05	0.01	2
	GO:0010324	membrane invagination	143	10	1.89	2.10E-05	0.0105	1.97881
	GO:0140014	mitotic nuclear division	286	14	3.77	2.90E-05	0.0145	1.83863
	GO:0045132	meiotic chromosome segregation	93	8	1.23	3.20E-05	0.016	1.79588
	GO:0006898	receptor-mediated endocytosis	328	15	4.33	3.30E-05	0.0165	1.78252
	GO:0050871	positive regulation of B cell activation	151	10	1.99	3.30E-05	0.0165	1.78252
	GO:0002460	adaptive immune response based on somati...	370	16	4.88	3.60E-05	0.018	1.74473
	GO:0006695	cholesterol biosynthetic process	75	7	0.99	6.00E-05	0.03	1.52288
	GO:1902653	secondary alcohol biosynthetic process	75	7	0.99	6.00E-05	0.03	1.52288
	GO:0002250	adaptive immune response	701	23	9.25	6.10E-05	0.0305	1.5157
	GO:0009617	response to bacterium	750	24	9.9	6.20E-05	0.031	1.50864
	GO:0008608	attachment of spindle microtubules to ki...	34	5	0.45	7.90E-05	0.0395	1.4034
	GO:0016126	sterol biosynthetic process	81	7	1.07	9.80E-05	0.049	1.3098
	GO:0006950	response to stress	4162	81	54.93	9.90E-05	0.0495	1.30539
	GO:0046890	regulation of lipid biosynthetic process	207	11	2.73	0.0001	0.05	1.30103
**Day3-Up**	GO:0061299	retina vasculature morphogenesis in came...	11	4	0.07	5.30E-07	0.00027	3.57675
	GO:0061298	retina vasculature development in camera...	18	4	0.12	4.70E-06	0.00235	2.62893
	GO:0043010	camera-type eye development	332	11	2.14	1.10E-05	0.0055	2.25964
	GO:0001654	eye development	384	11	2.48	4.00E-05	0.02	1.69897
	GO:0150063	visual system development	388	11	2.5	4.40E-05	0.022	1.65758
	GO:0048880	sensory system development	394	11	2.54	5.10E-05	0.0255	1.59346
	GO:0060041	retina development in camera-type eye	150	7	0.97	5.40E-05	0.027	1.56864
	GO:0060856	establishment of blood-brain barrier	12	3	0.08	5.50E-05	0.0275	1.56067
**Day3-Down**	GO:0006707	cholesterol catabolic process	11	4	0.11	2.90E-06	0.00145	2.83863
	GO:0016127	sterol catabolic process	11	4	0.11	2.90E-06	0.00145	2.83863
	GO:0006706	steroid catabolic process	27	5	0.27	6.00E-06	0.003	2.52288
	GO:0007417	central nervous system development	1042	25	10.29	3.70E-05	0.0185	1.73283
**Day5-Up**	GO:0034765	regulation of ion transmembrane transpor...	489	15	4.54	5.50E-05	0.0275	1.56067
	GO:0034762	regulation of transmembrane transport	577	16	5.36	0.0001	0.05	1.30103
**Day5-Down**	GO:0006941	striated muscle contraction	178	12	2.93	4.10E-05	0.0205	1.68825
	GO:0007517	muscle organ development	407	19	6.7	5.00E-05	0.025	1.60206

Table S6 GO terms (BP) for k-means cluster

	GO.ID	Term	Annotated	Significa	Expected	pvalue	FDR	score
	GO:0045653	negative regulation of megakaryocyte dif...	18	14	0.23	8.60E-24	4.30E-21	20.3665
	GO:0006335	DNA replication-dependent nucleosome ass...	32	14	0.42	1.10E-18	5.50E-16	15.2596
	GO:0034723	DNA replication-dependent nucleosome org...	32	14	0.42	1.10E-18	5.50E-16	15.2596
	GO:0000183	rDNA heterochromatin assembly	40	14	0.52	5.10E-17	2.55E-14	13.5935
	GO:0034080	CENP-A containing nucleosome assembly	43	14	0.56	1.70E-16	8.50E-14	13.0706
	GO:0061641	CENP-A containing chromatin organization	43	14	0.56	1.70E-16	8.50E-14	13.0706
	GO:0031055	chromatin remodeling at centromere	47	14	0.61	6.90E-16	3.45E-13	12.4622
	GO:0006336	DNA replication-independent nucleosome a...	53	14	0.69	4.50E-15	2.25E-12	11.6478
	GO:0034724	DNA replication-independent nucleosome o...	54	14	0.7	6.00E-15	3.00E-12	11.5229
	GO:0016233	telomere capping	55	14	0.72	8.00E-15	4.00E-12	11.3979
	GO:0034508	centromere complex assembly	56	14	0.73	1.10E-14	5.50E-12	11.2596
	GO:0031507	heterochromatin assembly	72	15	0.94	2.20E-14	1.10E-11	10.9586
	GO:0043486	histone exchange	59	14	0.77	2.30E-14	1.15E-11	10.9393
	GO:0070828	heterochromatin organization	80	15	1.04	1.10E-13	5.50E-11	10.2596
	GO:0045652	regulation of megakaryocyte differentiat...	82	14	1.07	3.00E-12	1.50E-09	8.82391
	GO:0043044	ATP-dependent chromatin remodeling	90	14	1.17	1.10E-11	5.50E-09	8.25964
	GO:0045638	negative regulation of myeloid cell diff...	95	14	1.24	2.40E-11	1.20E-08	7.92082
	GO:0006303	double-strand break repair via nonhomolo...	96	14	1.25	2.70E-11	1.35E-08	7.86967
	GO:0030219	megakaryocyte differentiation	100	14	1.31	4.80E-11	2.40E-08	7.61979
	GO:0000726	non-recombinational repair	104	14	1.36	8.30E-11	4.15E-08	7.38195
	GO:0060964	regulation of gene silencing by miRNA	116	14	1.51	3.70E-10	1.85E-07	6.73283
**Cluster 1**	GO:0060147	regulation of posttranscriptional gene s...	120	14	1.57	5.80E-10	2.90E-07	6.5376
	GO:0060966	regulation of gene silencing by RNA	120	14	1.57	5.80E-10	2.90E-07	6.5376
	GO:0097549	chromatin organization involved in negat...	143	15	1.87	6.30E-10	3.15E-07	6.50169
	GO:0045814	negative regulation of gene expression, ...	126	14	1.64	1.10E-09	5.50E-07	6.25964
	GO:0034401	chromatin organization involved in regul...	156	15	2.04	2.10E-09	1.05E-06	5.97881
	GO:0060968	regulation of gene silencing	140	14	1.83	4.50E-09	2.25E-06	5.64782
	GO:0006334	nucleosome assembly	145	14	1.89	7.00E-09	3.50E-06	5.45593
	GO:1903707	negative regulation of hemopoiesis	158	14	2.06	2.10E-08	1.05E-05	4.97881
	GO:0000723	telomere maintenance	161	14	2.1	2.70E-08	1.35E-05	4.86967
	GO:0031497	chromatin assembly	195	15	2.55	4.50E-08	2.25E-05	4.64782
	GO:0032200	telomere organization	174	14	2.27	7.20E-08	3.60E-05	4.4437
	GO:0040029	regulation of gene expression, epigeneti...	209	15	2.73	1.10E-07	5.50E-05	4.25964
	GO:0034728	nucleosome organization	183	14	2.39	1.40E-07	7.00E-05	4.1549
	GO:0006338	chromatin remodeling	218	15	2.85	1.90E-07	9.50E-05	4.02228
	GO:0006333	chromatin assembly or disassembly	220	15	2.87	2.20E-07	0.00011	3.95861
	GO:0006302	double-strand break repair	262	16	3.42	3.80E-07	0.00019	3.72125
	GO:0006323	DNA packaging	240	15	3.13	6.60E-07	0.00033	3.48149
	GO:0006352	DNA-templated transcription, initiation	249	15	3.25	1.10E-06	0.00055	3.25964
	GO:0065004	protein-DNA complex assembly	243	14	3.17	4.10E-06	0.00205	2.68825
	GO:0071824	protein-DNA complex subunit organization	282	15	3.68	4.90E-06	0.00245	2.61083
	GO:0045637	regulation of myeloid cell differentiati...	263	14	3.43	1.00E-05	0.005	2.30103
	GO:0071103	DNA conformation change	355	16	4.63	1.90E-05	0.0095	2.02228
	GO:0098813	nuclear chromosome segregation	272	15	2.09	3.00E-09	1.50E-06	5.82391
	GO:0000819	sister chromatid segregation	196	13	1.5	3.90E-09	1.95E-06	5.70997
	GO:0000070	mitotic sister chromatid segregation	161	12	1.24	4.30E-09	2.15E-06	5.66756
	GO:0007059	chromosome segregation	334	16	2.56	6.60E-09	3.30E-06	5.48149
	GO:0140014	mitotic nuclear division	286	14	2.19	4.60E-08	2.30E-05	4.63827
	GO:0051983	regulation of chromosome segregation	107	9	0.82	1.40E-07	7.00E-05	4.1549
	GO:0000280	nuclear division	428	16	3.28	2.00E-07	1.00E-04	4
	GO:0033045	regulation of sister chromatid segregati...	84	8	0.64	2.70E-07	0.00014	3.86967
	GO:0045132	meiotic chromosome segregation	93	8	0.71	5.90E-07	0.0003	3.53018
	GO:0051321	meiotic cell cycle	254	12	1.95	6.40E-07	0.00032	3.49485
	GO:0048285	organelle fission	476	16	3.65	8.40E-07	0.00042	3.37675
	GO:0000278	mitotic cell cycle	1053	24	8.08	1.70E-06	0.00085	3.07058
	GO:1903047	mitotic cell cycle process	905	22	6.95	1.70E-06	0.00085	3.07058
	GO:1903046	meiotic cell cycle process	193	10	1.48	2.50E-06	0.00125	2.90309
	GO:0051301	cell division	603	17	4.63	4.10E-06	0.00205	2.68825
	GO:1902652	secondary alcohol metabolic process	162	9	1.24	4.60E-06	0.0023	2.63827
	GO:0016043	cellular component organization	6526	77	50.08	5.50E-06	0.00275	2.56067
	GO:0008608	attachment of spindle microtubules to ki...	34	5	0.26	5.80E-06	0.0029	2.5376
	GO:0010965	regulation of mitotic sister chromatid s...	61	6	0.47	7.30E-06	0.00365	2.43771
	GO:0051310	metaphase plate congression	61	6	0.47	7.30E-06	0.00365	2.43771
	GO:0051304	chromosome separation	94	7	0.72	8.00E-06	0.004	2.39794
	GO:0140013	meiotic nuclear division	177	9	1.36	9.40E-06	0.0047	2.3279
	GO:0051306	mitotic sister chromatid separation	64	6	0.49	9.60E-06	0.0048	2.31876
**Cluster 2**	GO:0022402	cell cycle process	1422	27	10.91	1.10E-05	0.0055	2.25964
	GO:1905818	regulation of chromosome separation	66	6	0.51	1.20E-05	0.006	2.22185
	GO:2000816	negative regulation of mitotic sister ch...	39	5	0.3	1.20E-05	0.006	2.22185
	GO:1905819	negative regulation of chromosome separa...	40	5	0.31	1.30E-05	0.0065	2.18709
	GO:0007049	cell cycle	1882	32	14.44	1.40E-05	0.007	2.1549
	GO:0070192	chromosome organization involved in meio...	70	6	0.54	1.60E-05	0.008	2.09691
	GO:0033048	negative regulation of mitotic sister ch...	42	5	0.32	1.70E-05	0.0085	2.07058
	GO:0071840	cellular component organization or bioge...	6717	77	51.55	1.70E-05	0.0085	2.07058
	GO:0033047	regulation of mitotic sister chromatid s...	72	6	0.55	1.90E-05	0.0095	2.02228
	GO:0033046	negative regulation of sister chromatid ...	44	5	0.34	2.10E-05	0.0105	1.97881
	GO:0051985	negative regulation of chromosome segreg...	45	5	0.35	2.40E-05	0.012	1.92082

	GO:0006695	cholesterol biosynthetic process	75	6	0.58	2.40E-05	0.012	1.92082
	GO:1902653	secondary alcohol biosynthetic process	75	6	0.58	2.40E-05	0.012	1.92082
	GO:0050000	chromosome localization	79	6	0.61	3.30E-05	0.0165	1.78252
	GO:0051303	establishment of chromosome localization	79	6	0.61	3.30E-05	0.0165	1.78252
	GO:0016126	sterol biosynthetic process	81	6	0.62	3.70E-05	0.0185	1.73283
	GO:0045839	negative regulation of mitotic nuclear d...	52	5	0.4	4.90E-05	0.0245	1.61083
	GO:0030071	regulation of mitotic metaphase/anaphase...	56	5	0.43	7.00E-05	0.035	1.45593
	GO:0007091	metaphase/anaphase transition of mitotic...	58	5	0.45	8.20E-05	0.041	1.38722
	GO:1902099	regulation of metaphase/anaphase transit...	58	5	0.45	8.20E-05	0.041	1.38722
	GO:0051276	chromosome organization	1253	23	9.62	8.70E-05	0.0435	1.36151
	GO:0044784	metaphase/anaphase transition of cell cy...	60	5	0.46	9.70E-05	0.0485	1.31426
	GO:0051784	negative regulation of nuclear division	60	5	0.46	9.70E-05	0.0485	1.31426
**Cluster 3**	GO:0002819	regulation of adaptive immune response	168	12	1.26	5.50E-09	2.75E-06	5.56067
	GO:0019883	antigen processing and presentation of e...	24	6	0.18	2.00E-08	1.00E-05	5
	GO:0002822	regulation of adaptive immune response b...	153	11	1.15	2.30E-08	1.15E-05	4.9393
	GO:0001913	T cell mediated cytotoxicity	44	7	0.33	3.60E-08	1.80E-05	4.74473
	GO:0002475	antigen processing and presentation via ...	14	5	0.11	4.30E-08	2.15E-05	4.66756
	GO:0002824	positive regulation of adaptive immune r...	105	9	0.79	1.00E-07	5.00E-05	4.30103
	GO:0002821	positive regulation of adaptive immune r...	110	9	0.83	1.50E-07	7.50E-05	4.12494
	GO:0001914	regulation of T cell mediated cytotoxici...	35	6	0.26	2.20E-07	0.00011	3.95861
	GO:0002706	regulation of lymphocyte mediated immuni...	154	10	1.16	2.60E-07	0.00013	3.88606
	GO:0002703	regulation of leukocyte mediated immunit...	211	11	1.59	6.00E-07	3.00E-04	3.52288
	GO:0001906	cell killing	172	10	1.29	7.40E-07	0.00037	3.4318
	GO:0002705	positive regulation of leukocyte mediate...	138	9	1.04	1.00E-06	5.00E-04	3.30103
	GO:0002456	T cell mediated immunity	104	8	0.78	1.20E-06	6.00E-04	3.22185
	GO:0002709	regulation of T cell mediated immunity	73	7	0.55	1.30E-06	0.00065	3.18709
	GO:0002708	positive regulation of lymphocyte mediat...	106	8	0.8	1.40E-06	7.00E-04	3.1549
	GO:0001910	regulation of leukocyte mediated cytotox...	75	7	0.56	1.50E-06	0.00075	3.12494
	GO:0001909	leukocyte mediated cytotoxicity	108	8	0.81	1.60E-06	8.00E-04	3.09691
	GO:0001916	positive regulation of T cell mediated c...	28	5	0.21	1.90E-06	0.00095	3.02228
	GO:0002711	positive regulation of T cell mediated i...	50	6	0.38	2.00E-06	0.001	3
	GO:0001912	positive regulation of leukocyte mediate...	52	6	0.39	2.50E-06	0.00125	2.90309
	GO:0002460	adaptive immune response based on somati...	370	13	2.79	4.70E-06	0.00235	2.62893
	GO:0031341	regulation of cell killing	92	7	0.69	6.10E-06	0.00305	2.5157
	GO:0031343	positive regulation of cell killing	61	6	0.46	6.50E-06	0.00325	2.48812
	GO:0032943	mononuclear cell proliferation	286	11	2.15	1.10E-05	0.0055	2.25964
	GO:0002697	regulation of immune effector process	470	14	3.54	1.30E-05	0.0065	2.18709
	GO:0007520	myoblast fusion	45	5	0.34	2.20E-05	0.011	1.95861
	GO:0051250	negative regulation of lymphocyte activa...	154	8	1.16	2.20E-05	0.011	1.95861
	GO:0002250	adaptive immune response	701	17	5.28	2.20E-05	0.011	1.95861
	GO:0050798	activated T cell proliferation	46	5	0.35	2.40E-05	0.012	1.92082
	GO:0070661	leukocyte proliferation	313	11	2.36	2.60E-05	0.013	1.88606
	GO:0050670	regulation of lymphocyte proliferation	219	9	1.65	4.30E-05	0.0215	1.66756
	GO:0032944	regulation of mononuclear cell prolifera...	221	9	1.66	4.70E-05	0.0235	1.62893
	GO:0002699	positive regulation of immune effector p...	223	9	1.68	5.00E-05	0.025	1.60206
	GO:0046651	lymphocyte proliferation	283	10	2.13	5.80E-05	0.029	1.5376
	GO:0019882	antigen processing and presentation	232	9	1.75	6.80E-05	0.034	1.46852
	GO:0000768	syncytium formation by plasma membrane f...	58	5	0.44	7.50E-05	0.0375	1.42597
	GO:0140253	cell-cell fusion	58	5	0.44	7.50E-05	0.0375	1.42597
	GO:0002695	negative regulation of leukocyte activat...	184	8	1.39	7.90E-05	0.0395	1.4034
	GO:0019884	antigen processing and presentation of e...	185	8	1.39	8.20E-05	0.041	1.38722
	GO:0006955	immune response	2306	34	17.36	8.70E-05	0.0435	1.36151
	GO:0070663	regulation of leukocyte proliferation	240	9	1.81	8.80E-05	0.044	1.35655
	GO:0006949	syncytium formation	60	5	0.45	8.90E-05	0.0445	1.35164
**Cluster 4**	GO:0038111	interleukin-7-mediated signaling pathway	30	10	0.34	6.50E-13	3.25E-10	9.48812
	GO:0006335	DNA replication-dependent nucleosome ass...	32	10	0.36	1.40E-12	7.00E-10	9.1549
	GO:0034723	DNA replication-dependent nucleosome org...	32	10	0.36	1.40E-12	7.00E-10	9.1549
	GO:0000183	rDNA heterochromatin assembly	40	10	0.45	1.70E-11	8.50E-09	8.07058
	GO:0098760	response to interleukin-7	42	10	0.47	2.90E-11	1.45E-08	7.83863
	GO:0098761	cellular response to interleukin-7	42	10	0.47	2.90E-11	1.45E-08	7.83863
	GO:0070828	heterochromatin organization	80	11	0.9	1.50E-09	7.50E-07	6.12494
	GO:0045652	regulation of megakaryocyte differentiat...	82	11	0.92	2.00E-09	1.00E-06	6
	GO:0031507	heterochromatin assembly	72	10	0.81	7.70E-09	3.85E-06	5.41454
	GO:0030219	megakaryocyte differentiation	100	11	1.12	1.70E-08	8.50E-06	5.07058
	GO:0060968	regulation of gene silencing	140	12	1.57	6.30E-08	3.15E-05	4.50169
	GO:0060964	regulation of gene silencing by miRNA	116	11	1.3	8.00E-08	4.00E-05	4.39794
	GO:0060147	regulation of posttranscriptional gene s...	120	11	1.35	1.10E-07	5.50E-05	4.25964
	GO:0060966	regulation of gene silencing by RNA	120	11	1.35	1.10E-07	5.50E-05	4.25964
	GO:0045814	negative regulation of gene expression, ...	126	11	1.42	1.90E-07	9.50E-05	4.02228
	GO:0097549	chromatin organization involved in negat...	143	11	1.61	6.70E-07	0.00034	3.47496
	GO:0006334	nucleosome assembly	145	11	1.63	7.70E-07	0.00039	3.41454
	GO:0034401	chromatin organization involved in regul...	156	11	1.75	1.60E-06	8.00E-04	3.09691
	GO:0034728	nucleosome organization	183	11	2.06	7.50E-06	0.00375	2.42597
	GO:0045637	regulation of myeloid cell differentiati...	263	13	2.96	9.40E-06	0.0047	2.3279
	GO:0031497	chromatin assembly	195	11	2.19	1.40E-05	0.007	2.1549
	GO:0006323	DNA packaging	240	12	2.7	1.90E-05	0.0095	2.02228
	GO:0040029	regulation of gene expression, epigeneti...	209	11	2.35	2.60E-05	0.013	1.88606
	GO:0032200	telomere organization	174	10	1.96	2.90E-05	0.0145	1.83863
	GO:0007596	blood coagulation	343	14	3.86	3.60E-05	0.018	1.74473

	GO:0006333	chromatin assembly or disassembly	220	11	2.47	4.10E-05	0.0205	1.68825
	GO:0007599	hemostasis	348	14	3.91	4.30E-05	0.0215	1.66756
	GO:0050817	coagulation	349	14	3.92	4.40E-05	0.022	1.65758
	GO:0065004	protein-DNA complex assembly	243	11	2.73	0.0001	0.05	1.30103
	GO:0050798	activated T cell proliferation	46	5	0.28	8.80E-06	0.0044	2.35655
	GO:0051250	negative regulation of lymphocyte activa...	154	7	0.94	4.50E-05	0.0225	1.64782
**Cluster 5**	GO:0032943	mononuclear cell proliferation	286	9	1.75	6.70E-05	0.0335	1.47496
	GO:0046007	negative regulation of activated T cell ...	14	3	0.09	7.70E-05	0.0385	1.41454
	GO:0050868	negative regulation of T cell activation	118	6	0.72	8.70E-05	0.0435	1.36151
	GO:0009611	response to wounding	656	18	5.29	6.20E-06	0.0031	2.50864
	GO:0042060	wound healing	536	16	4.32	7.40E-06	0.0037	2.4318
	GO:0002576	platelet degranulation	129	8	1.04	1.00E-05	0.005	2.30103
	GO:0019722	calcium-mediated signaling	222	10	1.79	1.30E-05	0.0065	2.18709
	GO:0019932	second-messenger-mediated signaling	456	14	3.68	2.10E-05	0.0105	1.97881
**Cluster 6**	GO:0007596	blood coagulation	343	12	2.77	2.30E-05	0.0115	1.9393
	GO:0035584	calcium-mediated signaling using intrace...	22	4	0.18	2.70E-05	0.0135	1.86967
	GO:0007599	hemostasis	348	12	2.81	2.70E-05	0.0135	1.86967
	GO:0050817	coagulation	349	12	2.81	2.80E-05	0.014	1.85387
	GO:1903036	positive regulation of response to wound...	74	6	0.6	3.00E-05	0.015	1.82391
	GO:0032501	multicellular organismal process	7970	89	64.28	6.40E-05	0.032	1.49485
	GO:0032496	response to lipopolysaccharide	334	11	2.69	8.80E-05	0.044	1.35655
	GO:0045653	negative regulation of megakaryocyte dif...	18	14	0.29	1.40E-22	7.00E-20	19.1549
	GO:0006335	DNA replication-dependent nucleosome ass...	32	14	0.51	1.70E-17	8.50E-15	14.0706
	GO:0034723	DNA replication-dependent nucleosome org...	32	14	0.51	1.70E-17	8.50E-15	14.0706
	GO:0000183	rDNA heterochromatin assembly	40	14	0.63	7.60E-16	3.80E-13	12.4202
	GO:0034080	CENP-A containing nucleosome assembly	43	14	0.68	2.40E-15	1.20E-12	11.9208
	GO:0061641	CENP-A containing chromatin organization	43	14	0.68	2.40E-15	1.20E-12	11.9208
	GO:0031055	chromatin remodeling at centromere	47	14	0.74	1.00E-14	5.00E-12	11.301
	GO:0006336	DNA replication-independent nucleosome a...	53	14	0.84	6.50E-14	3.25E-11	10.4881
	GO:0034724	DNA replication-independent nucleosome o...	54	14	0.86	8.70E-14	4.35E-11	10.3615
	GO:0016233	telomere capping	55	14	0.87	1.10E-13	5.50E-11	10.2596
	GO:0034508	centromere complex assembly	56	14	0.89	1.50E-13	7.50E-11	10.1249
	GO:0043486	histone exchange	59	14	0.93	3.30E-13	1.65E-10	9.78252
	GO:0031507	heterochromatin assembly	72	14	1.14	6.20E-12	3.10E-09	8.50864
	GO:0045814	negative regulation of gene expression, ...	126	17	2	1.60E-11	8.00E-09	8.09691
	GO:0070828	heterochromatin organization	80	14	1.27	2.80E-11	1.40E-08	7.85387
	GO:0045652	regulation of megakaryocyte differentiat...	82	14	1.3	3.90E-11	1.95E-08	7.70997
	GO:0097549	chromatin organization involved in negat...	143	17	2.26	1.20E-10	6.00E-08	7.22185
	GO:0043044	ATP-dependent chromatin remodeling	90	14	1.43	1.40E-10	7.00E-08	7.1549
	GO:0045638	negative regulation of myeloid cell diff...	95	14	1.5	3.00E-10	1.50E-07	6.82391
	GO:0006303	double-strand break repair via nonhomolo...	96	14	1.52	3.50E-10	1.75E-07	6.75696
**Cluster 7**	GO:0034401	chromatin organization involved in regul...	156	17	2.47	4.90E-10	2.45E-07	6.61083
	GO:0030219	megakaryocyte differentiation	100	14	1.58	6.10E-10	3.05E-07	6.5157
	GO:0000726	non-recombinational repair	104	14	1.65	1.00E-09	5.00E-07	6.30103
	GO:0006334	nucleosome assembly	145	16	2.3	1.30E-09	6.50E-07	6.18709
	GO:0060964	regulation of gene silencing by miRNA	116	14	1.84	4.50E-09	2.25E-06	5.64782
	GO:0060968	regulation of gene silencing	140	15	2.22	6.60E-09	3.30E-06	5.48149
	GO:0040029	regulation of gene expression, epigeneti...	209	18	3.31	6.80E-09	3.40E-06	5.46852
	GO:0060147	regulation of posttranscriptional gene s...	120	14	1.9	7.00E-09	3.50E-06	5.45593
	GO:0060966	regulation of gene silencing by RNA	120	14	1.9	7.00E-09	3.50E-06	5.45593
	GO:1903707	negative regulation of hemopoiesis	158	15	2.5	3.40E-08	1.70E-05	4.76955
	GO:0034728	nucleosome organization	183	16	2.9	3.80E-08	1.90E-05	4.72125
	GO:0031497	chromatin assembly	195	16	3.09	9.30E-08	4.65E-05	4.33255
	GO:0000723	telomere maintenance	161	14	2.55	2.90E-07	0.00015	3.83863
	GO:0006333	chromatin assembly or disassembly	220	16	3.48	4.80E-07	0.00024	3.61979
	GO:0032200	telomere organization	174	14	2.76	7.60E-07	0.00038	3.42022
	GO:0006323	DNA packaging	240	16	3.8	1.50E-06	0.00075	3.12494
	GO:0065004	protein-DNA complex assembly	243	16	3.85	1.80E-06	9.00E-04	3.04576
	GO:0006338	chromatin remodeling	218	14	3.45	1.10E-05	0.0055	2.25964
	GO:0006352	DNA-templated transcription, initiation	249	15	3.94	1.10E-05	0.0055	2.25964
	GO:0071824	protein-DNA complex subunit organization	282	16	4.47	1.20E-05	0.006	2.22185
	GO:0006302	double-strand break repair	262	15	4.15	2.00E-05	0.01	2
	GO:0045637	regulation of myeloid cell differentiati...	263	15	4.17	2.10E-05	0.0105	1.97881
	GO:0002475	antigen processing and presentation via ...	14	5	0.08	1.10E-08	5.50E-06	5.25964
	GO:0019883	antigen processing and presentation of e...	24	5	0.14	2.30E-07	0.00012	3.9393
	GO:0001916	positive regulation of T cell mediated c...	28	5	0.16	5.20E-07	0.00026	3.58503
	GO:0001912	positive regulation of leukocyte mediate...	52	6	0.3	5.30E-07	0.00027	3.57675
	GO:0002705	positive regulation of leukocyte mediate...	138	8	0.8	1.40E-06	7.00E-04	3.1549
	GO:0031343	positive regulation of cell killing	61	6	0.35	1.40E-06	7.00E-04	3.1549
	GO:0001914	regulation of T cell mediated cytotoxici...	35	5	0.2	1.70E-06	0.00085	3.07058
	GO:0002824	positive regulation of adaptive immune r...	105	7	0.61	2.50E-06	0.00125	2.90309
	GO:0002708	positive regulation of lymphocyte mediat...	106	7	0.61	2.70E-06	0.00135	2.86967
	GO:0001909	leukocyte mediated cytotoxicity	108	7	0.62	3.10E-06	0.00155	2.80967
	GO:0002821	positive regulation of adaptive immune r...	110	7	0.63	3.50E-06	0.00175	2.75696
**Cluster 8**	GO:0001910	regulation of leukocyte mediated cytotox...	75	6	0.43	4.70E-06	0.00235	2.62893
	GO:0001913	T cell mediated cytotoxicity	44	5	0.25	5.30E-06	0.00265	2.57675
	GO:0001906	cell killing	172	8	0.99	7.10E-06	0.00355	2.44977
	GO:0002711	positive regulation of T cell mediated i...	50	5	0.29	1.00E-05	0.005	2.30103
	GO:0031341	regulation of cell killing	92	6	0.53	1.50E-05	0.0075	2.12494

	GO:0002822	regulation of adaptive immune response b...	153	7	0.88	3.00E-05	0.015	1.82391
	GO:0002703	regulation of leukocyte mediated immunit...	211	8	1.22	3.10E-05	0.0155	1.80967
	GO:0002706	regulation of lymphocyte mediated immuni...	154	7	0.89	3.10E-05	0.0155	1.80967
	GO:0002699	positive regulation of immune effector p...	223	8	1.29	4.60E-05	0.023	1.63827
	GO:0002819	regulation of adaptive immune response	168	7	0.97	5.50E-05	0.0275	1.56067
	GO:0002709	regulation of T cell mediated immunity	73	5	0.42	6.40E-05	0.032	1.49485
	GO:0051049	regulation of transport	1821	24	10.5	9.80E-05	0.049	1.3098
	GO:0061299	retina vasculature morphogenesis in came...	11	3	0.04	9.30E-06	0.00465	2.33255
	GO:0060856	establishment of blood-brain barrier	12	3	0.05	1.20E-05	0.006	2.22185
	GO:0042127	regulation of cell population proliferat...	1766	19	6.91	3.80E-05	0.019	1.72125
**Cluster 9**	GO:0061298	retina vasculature development in camera...	18	3	0.07	4.50E-05	0.0225	1.64782
	GO:0043010	camera-type eye development	332	8	1.3	4.60E-05	0.023	1.63827
	GO:0008283	cell population proliferation	2047	20	8.01	8.90E-05	0.0445	1.35164
	GO:0060479	lung cell differentiation	23	3	0.09	9.60E-05	0.048	1.31876

Table S7 KEGG pathway for k-means cluster

	KEGGID	Pvalue	OddsRatio	ExpCount	Count	Size	Term
**Cluster 1**	5322	2.1E-15	29.4834711	0.90389	15	136	Systemic lupus erythematosus
	860	0.03275	7.63085036	0.28579	2	43	Porphyrin and chlorophyll metabolism
	4623	0.05293	5.78078078	0.37219	2	56	Cytosolic DNA-sensing pathway
	140	0.05293	5.78078078	0.37219	2	56	Steroid hormone biosynthesis
**Cluster 2**	100	4.2E-09	67.0269231	0.14894	6	19	Steroid biosynthesis
	4114	0.0017	6.5135628	0.87798	5	112	Oocyte meiosis
	5200	0.0126	3.09629451	2.55556	7	326	Pathways in cancer
	4110	0.01564	4.52539683	0.97205	4	124	Cell cycle
	4520	0.01913	5.73289037	0.57226	3	73	Adherens junction
	4970	0.03204	4.65332612	0.69768	3	89	Salivary secretion
	5215	0.03204	4.65332612	0.69768	3	89	Prostate cancer
	5110	0.06653	5.04370629	0.42331	2	54	Vibrio cholerae infection
	3450	0.09735	10.7592593	0.10191	1	13	Non-homologous end-joining
	4115	0.09894	3.96418733	0.53306	2	68	p53 signaling pathway
**Cluster 3**	780	0.00715	292.3	0.00716	1	2	Biotin metabolism
	4930	0.01246	13.2745995	0.17178	2	48	Type II diabetes mellitus
	4122	0.03524	32.4333333	0.03579	1	10	Sulfur relay system
	5323	0.04127	6.8101715	0.32566	2	91	Rheumatoid arthritis
	4666	0.04377	6.58466819	0.3364	2	94	Fc gamma R-mediated phagocytosis
	120	0.05582	19.44	0.05726	1	16	Primary bile acid biosynthesis
	410	0.07597	13.8714286	0.07873	1	22	beta-Alanine metabolism
	4530	0.07986	4.6291498	0.47239	2	132	Tight junction
	5160	0.08196	4.55741627	0.47955	2	134	Hepatitis C
	4977	0.0826	12.6608696	0.08589	1	24	Vitamin digestion and absorption
	563	0.0859	12.13125	0.08947	1	25	Glycosylphosphatidylinositol(GPI)-anchor biosynthesis
	53	0.08918	11.644	0.09305	1	26	Ascorbate and aldarate metabolism
	4966	0.09246	11.1942308	0.09663	1	27	Collecting duct acid secretion
	340	0.09897	10.3910714	0.10378	1	29	Histidine metabolism
**Cluster 4**	5322	8E-09	11.4596774	1.39059	12	136	Systemic lupus erythematosus
	5146	0.00068	6.34222222	1.08384	6	106	Amoebiasis
	4520	0.00083	7.67379679	0.74642	5	73	Adherens junction
	5215	0.00203	6.19480519	0.91002	5	89	Prostate cancer
	5213	0.01575	6.1858217	0.5317	3	52	Endometrial cancer
	4620	0.01995	4.16180758	1.04294	4	102	Toll-like receptor signaling pathway
	5221	0.02012	5.60818713	0.58282	3	57	Acute myeloid leukemia
	410	0.02083	9.97931034	0.22495	2	22	beta-Alanine metabolism
	4810	0.02087	3.00644122	2.17791	6	213	Regulation of actin cytoskeleton
	5142	0.02127	4.07714286	1.06339	4	104	Chagas disease (American trypanosomiasis)
	5210	0.02509	5.12845674	0.63395	3	62	Colorectal cancer
	5211	0.0343	4.50981932	0.71575	3	70	Renal cell carcinoma
	5220	0.03815	4.31428571	0.74642	3	73	Chronic myeloid leukemia
	5412	0.03948	4.25277984	0.75665	3	74	Arrhythmogenic right ventricular cardiomyopathy (ARVC)
	250	0.04191	6.64137931	0.3272	2	32	Alanine, aspartate and glutamate metabolism
	4062	0.04306	2.77865613	1.93252	5	189	Chemokine signaling pathway
	5020	0.04931	6.03448276	0.35787	2	35	Prion diseases
	5222	0.05563	3.67522465	0.86912	3	85	Small cell lung cancer
	4012	0.05886	3.58646617	0.88957	3	87	ErbB signaling pathway
	4640	0.06051	3.54365325	0.8998	3	88	Hematopoietic cell lineage
	5323	0.06559	3.42105263	0.93047	3	91	Rheumatoid arthritis
	4960	0.06821	4.97241379	0.42945	2	42	Aldosterone-regulated sodium reabsorption
	4666	0.07085	3.30653557	0.96115	3	94	Fc gamma R-mediated phagocytosis
	4630	0.07316	2.67596973	1.58487	4	155	Jak-STAT signaling pathway
	4973	0.07399	4.73399015	0.4499	2	44	Carbohydrate digestion and absorption
	4930	0.08599	4.31934033	0.4908	2	48	Type II diabetes mellitus
	430	0.09774	10.920904	0.10225	1	10	Taurine and hypotaurine metabolism
	4150	0.09854	3.97103448	0.5317	2	52	mTOR signaling pathway
**Cluster 5**	4060	0.00269	4.96911197	1.44513	6	265	Cytokine-cytokine receptor interaction
	5143	0.01533	11.7232323	0.19087	2	35	African trypanosomiasis
	4620	0.01755	5.99477534	0.55624	3	102	Toll-like receptor signaling pathway
	5150	0.03576	7.27421384	0.29993	2	55	Staphylococcus aureus infection
	4623	0.03696	7.1382716	0.30539	2	56	Cytosolic DNA-sensing pathway
	232	0.03757	31.344086	0.03817	1	7	Caffeine metabolism
	4621	0.0394	6.88095238	0.31629	2	58	NOD-like receptor signaling pathway
	4146	0.06846	4.98614719	0.43081	2	79	Peroxisome
	4640	0.08255	4.45736434	0.47989	2	88	Hematopoietic cell lineage
	120	0.08388	12.5182796	0.08725	1	16	Primary bile acid biosynthesis
	4614	0.08889	11.733871	0.09271	1	17	Renin-angiotensin system
	630	0.09387	11.0417457	0.09816	1	18	Glyoxylate and dicarboxylate metabolism
**Cluster 6**	260	0.00016	17.2261905	0.28357	4	32	Glycine, serine and threonine metabolism
	330	0.01185	6.92076831	0.47853	3	54	Arginine and proline metabolism
	4020	0.01952	3.49084612	1.56851	5	177	Calcium signaling pathway
	5412	0.02737	4.95400977	0.65576	3	74	Arrhythmogenic right ventricular cardiomyopathy (ARVC)
	4510	0.03109	3.06655756	1.77232	5	200	Focal adhesion
	5410	0.03671	4.38979592	0.73551	3	83	Hypertrophic cardiomyopathy (HCM)
	4512	0.03898	4.28123444	0.75324	3	85	ECM-receptor interaction
	5414	0.04497	4.03166784	0.79755	3	90	Dilated cardiomyopathy
	750	0.05203	22.7882353	0.05317	1	6	Vitamin B6 metabolism

	4960	0.05295	5.776	0.37219	2	42	Aldosterone-regulated sodium reabsorption
	4972	0.05965	3.57226156	0.89502	3	101	Pancreatic secretion
	5144	0.07459	4.7077551	0.45194	2	51	Malaria
	4270	0.08283	3.0899404	1.02795	3	116	Vascular smooth muscle contraction
**Cluster 7**	5322	7.1E-15	19.3537415	1.36742	17	136	Systemic lupus erythematosus
	4060	0.00118	3.90445313	2.66445	9	265	Cytokine-cytokine receptor interaction
	5320	0.01506	6.29737609	0.52284	3	52	Autoimmune thyroid disease
	4920	0.03051	4.73406593	0.68371	3	68	Adipocytokine signaling pathway
	5211	0.03286	4.59115139	0.70382	3	70	Renal cell carcinoma
	4350	0.05187	3.78835979	0.84458	3	84	TGF-beta signaling pathway
	5330	0.05287	5.78847118	0.37202	2	37	Allograft rejection
	5323	0.06299	3.48275162	0.91496	3	91	Rheumatoid arthritis
	5332	0.06347	5.19118309	0.41224	2	41	Graft-versus-host disease
	130	0.06833	16.6752874	0.07038	1	7	Ubiquinone and other terpenoid-quinone biosynthesis
	4940	0.06902	4.93624305	0.43234	2	43	Type I diabetes mellitus
**Cluster 8**	4640	0.00037	9.35037447	0.62986	5	88	Hematopoietic cell lineage
	980	0.00156	9.0479183	0.50818	4	71	Metabolism of xenobiotics by cytochrome P450
	982	0.00173	8.7826087	0.52249	4	73	Drug metabolism - cytochrome P450
	480	0.00529	9.45826514	0.35787	3	50	Glutathione metabolism
	4977	0.01249	13.1909091	0.17178	2	24	Vitamin digestion and absorption
	565	0.02702	8.51764706	0.25767	2	36	Ether lipid metabolism
	2010	0.03915	6.88571429	0.31493	2	44	ABC transporters
	5144	0.05114	5.89489796	0.36503	2	51	Malaria
	5150	0.05851	5.44622642	0.39366	2	55	Staphylococcus aureus infection
	4360	0.06405	3.47985348	0.92331	3	129	Axon guidance
	590	0.06623	5.06052632	0.42229	2	59	Arachidonic acid metabolism
	4514	0.06889	3.3704142	0.95194	3	133	Cell adhesion molecules (CAMs)
	5322	0.07264	3.29265471	0.97342	3	136	Systemic lupus erythematosus
	4610	0.08688	4.29776119	0.49387	2	69	Complement and coagulation cascades
	5412	0.09784	3.99583333	0.52965	2	74	Arrhythmogenic right ventricular cardiomyopathy (ARVC)
**Cluster 9**	5217	0.01471	12.148847	0.18746	2	55	Basal cell carcinoma
	5412	0.02574	8.91358025	0.25222	2	74	Arrhythmogenic right ventricular cardiomyopathy (ARVC)
	4916	0.04557	6.45230079	0.34424	2	101	Melanogenesis
	4310	0.09138	4.27927928	0.51125	2	150	Wnt signaling pathway
	5216	0.09448	10.9398496	0.09884	1	29	Thyroid cancer
	4630	0.09662	4.13580247	0.52829	2	155	Jak-STAT signaling pathway

Table S8 GSEA for SLC2A1 and STIMATE

Gene	Description	setSize	enrichmentScore	NES	pvalue	p.adjust	qvalues	rank	leading_edge	core_enrichment	
	KEGG_RIBOSOME	87	-0.694899981	-4.46363	1E-10	1.1E-08	7E-09	4253	tags=83%	list=19%	signal=67%
	KEGG_OXIDATIVE_PHOSPHORYLATION	108	-0.417004036	-2.78925	1.2E-10	1.1E-08	7E-09	4547	tags=47%	list=21%	signal=38%
	KEGG_PARKINSONS_DISEASE	105	-0.414384365	-2.76099	2.5E-10	1.5E-08	9.9E-09	4346	tags=47%	list=20%	signal=38%
	KEGG_HUNTINGTONS_DISEASE	163	-0.324668668	-2.38661	3E-08	1.4E-06	8.9E-07	4346	tags=39%	list=20%	signal=31%
	KEGG_REGULATION_OF_ACTIN_CYTOSKELETON	192	0.327094585	2.16559	1.9E-07	7.1E-06	4.6E-06	8089	tags=56%	list=37%	signal=36%
	KEGG_ADHERENS_JUNCTION	71	0.433873271	2.35995	2E-06	6.2E-05	4E-05	6550	tags=56%	list=30%	signal=40%
	KEGG_FOCAL_ADHESION	186	0.301626147	1.98954	7.1E-06	0.00019	0.00012	5439	tags=40%	list=25%	signal=30%
	KEGG_ALZHEIMERS_DISEASE	148	-0.288040374	-2.07479	1.2E-05	0.00028	0.00018	4145	tags=34%	list=19%	signal=28%
	KEGG_AMINOACYL_TRNA_BIOSYNTHESIS	22	-0.590800126	-2.58547	1.9E-05	0.00038	0.00024	7116	tags=73%	list=32%	signal=49%
	KEGG_LEUKOCYTE_TRANSENDOTHELIAL_MIGRATION	105	0.347816712	2.05356	2.3E-05	0.00041	0.00027	8735	tags=58%	list=40%	signal=35%
	KEGG_VALINE_LEUCINE_AND_ISOLEUCINE_DEGRADATION	43	-0.427891016	-2.24319	9.2E-05	0.00153	0.00099	8834	tags=67%	list=40%	signal=40%
	KEGG_SPLICEOSOME	126	-0.2768878	-1.90897	0.0001	0.0016	0.00103	4422	tags=33%	list=20%	signal=27%
	KEGG_ECM_RECEPTOR_INTERACTION	74	0.383968628	2.11836	0.00012	0.00174	0.00112	5566	tags=47%	list=25%	signal=35%
	KEGG_NEUROACTIVE_LIGAND_RECEPTOR_INTERACTION	180	0.282483051	1.8565	0.00016	0.00214	0.00138	6621	tags=46%	list=30%	signal=32%
	KEGG_HISTIDINE_METABOLISM	25	-0.502545499	-2.2939	0.00024	0.00289	0.00186	8103	tags=76%	list=37%	signal=48%
	KEGG_GAP_JUNCTION	77	0.356099313	1.98049	0.0003	0.00349	0.00225	5562	tags=44%	list=25%	signal=33%
	KEGG_PROGESTERONE_MEDIATED_OOCYTE_MATURATION	80	0.338251702	1.89721	0.00045	0.00462	0.00298	4887	tags=42%	list=22%	signal=33%
	KEGG_CELL_CYCLE	124	0.301412734	1.85409	0.00045	0.00462	0.00298	6077	tags=43%	list=28%	signal=31%
SLC2A1	KEGG_PATHOGENIC_ESCHERICHIA_COLI_INFECTION	52	0.389352863	1.96899	0.00062	0.00599	0.00386	5862	tags=46%	list=27%	signal=34%
	KEGG_DRUG_METABOLISM_CYTOCHROME_P450	39	-0.39925512	-2.03016	0.00065	0.00599	0.00386	7930	tags=64%	list=36%	signal=41%
	KEGG_ANTIGEN_PROCESSING_AND_PRESENTATION	69	-0.315226346	-1.88746	0.00115	0.01006	0.00648	4371	tags=38%	list=20%	signal=30%
	KEGG_INTESTINAL_IMMUNE_NETWORK_FOR_IGA_PRODUCTION	44	-0.365040818	-1.93017	0.00166	0.01384	0.00891	6849	tags=59%	list=31%	signal=41%
	KEGG_BASAL_TRANSCRIPTION_FACTORS	33	-0.405205346	-1.99809	0.00198	0.01579	0.01017	4306	tags=45%	list=20%	signal=37%
	KEGG_OLFACTORY_TRANSDUCTION	98	0.305140217	1.77816	0.00235	0.01794	0.01156	5414	tags=40%	list=25%	signal=30%
	KEGG_TYROSINE_METABOLISM	33	-0.397319548	-1.95921	0.00255	0.01865	0.01202	6347	tags=55%	list=29%	signal=39%
	KEGG_ALLOGRAFT_REJECTION	34	-0.394971482	-1.96736	0.00284	0.01999	0.01288	6254	tags=71%	list=28%	signal=51%
	KEGG_ASCORBATE_AND_ALDARATE_METABOLISM	11	-0.609478759	-2.07068	0.00576	0.03907	0.02517	8598	tags=100%	list=39%	signal=61%
	KEGG_TGF_BETA_SIGNALING_PATHWAY	80	0.2936355	1.64696	0.00614	0.04012	0.02585	4768	tags=36%	list=22%	signal=28%
	KEGG_PENTOSE_AND_GLUCURONATE_INTERCONVERSIONS	14	-0.538980289	-1.9789	0.00699	0.04313	0.02779	6580	tags=71%	list=30%	signal=50%
	KEGG_CYTOKINE_CYTOKINE_RECEPTOR_INTERACTION	220	0.230504812	1.56046	0.00742	0.04313	0.02779	6131	tags=37%	list=28%	signal=27%
	KEGG_VASCULAR_SMOOTH_MUSCLE_CONTRACTION	95	0.283440826	1.6398	0.00747	0.04313	0.02779	5414	tags=37%	list=25%	signal=28%
	KEGG_DILATED_CARDIOMYOPATHY	81	0.298588538	1.68233	0.00767	0.04313	0.02779	5439	tags=38%	list=25%	signal=29%
	KEGG_NUCLEOTIDE_EXCISION_REPAIR	44	-0.329203665	-1.74068	0.00778	0.04313	0.02779	4654	tags=41%	list=21%	signal=32%
	KEGG_PROSTATE_CANCER	87	0.286386489	1.62235	0.00853	0.04482	0.02887	5770	tags=40%	list=26%	signal=30%
	KEGG_STARCH_AND_SUCROSE_METABOLISM	29	0.403404111	1.72492	0.00873	0.04482	0.02887	4651	tags=48%	list=21%	signal=38%
	KEGG_AXON_GUIDANCE	113	0.270002658	1.62026	0.00882	0.04482	0.02887	6014	tags=42%	list=27%	signal=30%
	KEGG_O_GLYCAN_BIOSYNTHESIS	26	0.432069787	1.79059	0.0093	0.04602	0.02965	6187	tags=62%	list=28%	signal=44%
	KEGG_RIBOSOME	87	-0.754839339	-4.34148	1E-10	1.8E-08	1.1E-08	3178	tags=82%	list=14%	signal=71%
	KEGG_INSULIN_SIGNALING_PATHWAY	124	0.381507621	2.36554	1.5E-08	1.3E-06	8.1E-07	6000	tags=53%	list=26%	signal=40%
	KEGG_ENDOCYTOSIS	176	0.343869187	2.27892	2.7E-08	1.4E-06	8.7E-07	4706	tags=41%	list=20%	signal=33%
	KEGG_FC_GAMMA_R_MEDIATED_PHAGOCYTOSIS	94	0.422760349	2.47849	3.1E-08	1.4E-06	8.7E-07	4664	tags=49%	list=20%	signal=39%
	KEGG_ACUTE_MYELOID_LEUKEMIA	57	0.500055676	2.57979	3.9E-08	1.4E-06	8.8E-07	5671	tags=61%	list=24%	signal=46%
	KEGG_CHEMOKINE_SIGNALING_PATHWAY	176	0.338351374	2.24235	5.6E-08	1.7E-06	1E-06	6745	tags=54%	list=29%	signal=39%
	KEGG_NEUROTROPHIN_SIGNALING_PATHWAY	119	0.382002644	2.35213	9.3E-08	2.4E-06	1.5E-06	5671	tags=48%	list=24%	signal=36%
	KEGG_TOLL_LIKE_RECEPTOR_SIGNALING_PATHWAY	89	0.408713001	2.35713	3.4E-07	7.7E-06	4.7E-06	4580	tags=48%	list=20%	signal=39%
	KEGG_REGULATION_OF_ACTIN_CYTOSKELETON	189	0.308011104	2.06331	5.9E-07	1.2E-05	7.3E-06	5551	tags=42%	list=24%	signal=32%
	KEGG_B_CELL_RECEPTOR_SIGNALING_PATHWAY	74	0.419565034	2.32021	2E-06	3.6E-05	2.2E-05	5671	tags=58%	list=24%	signal=44%
	KEGG_LYSOSOME	117	0.357676115	2.19	2.2E-06	3.7E-05	2.2E-05	6949	tags=57%	list=30%	signal=40%
	KEGG_NOD_LIKE_RECEPTOR_SIGNALING_PATHWAY	59	0.455730416	2.37066	2.6E-06	3.9E-05	2.4E-05	4393	tags=53%	list=19%	signal=43%
	KEGG_EPITHELIAL_CELL_SIGNALING_IN_HELICOBACTER_PYLORI_INFECTION	61	0.438768664	2.33346	6.3E-06	8.9E-05	5.4E-05	4304	tags=51%	list=19%	signal=41%
	KEGG_PROTEASOME	44	0.476419222	2.30338	1.1E-05	0.00014	8.8E-05	5741	tags=57%	list=25%	signal=43%
	KEGG_MAPK_SIGNALING_PATHWAY	235	0.26477759	1.83833	1.4E-05	0.00017	0.00011	5671	tags=39%	list=24%	signal=30%
	KEGG_RENAL_CELL_CARCINOMA	70	0.404022664	2.19818	2.4E-05	0.00028	0.00017	5671	tags=49%	list=24%	signal=37%
	KEGG_APOPTOSIS	84	0.363315592	2.05365	3.8E-05	0.00041	0.00025	5909	tags=51%	list=26%	signal=38%
	KEGG_CHRONIC_MYELOID_LEUKEMIA	72	0.37301141	2.04057	8.2E-05	0.00083	0.00051	6479	tags=57%	list=28%	signal=41%
	KEGG_PATHWAYS_IN_CANCER	300	0.234429911	1.69699	8.7E-05	0.00084	0.00051	6479	tags=39%	list=28%	signal=29%
	KEGG_JAK_STAT_SIGNALING_PATHWAY	128	0.302292059	1.89631	0.00011	0.00098	0.0006	6584	tags=48%	list=28%	signal=34%
	KEGG_NATURAL_KILLER_CELL_MEDIATED_CYTOTOXICITY	113	0.329885963	2.00448	0.00013	0.00114	0.00069	5551	tags=48%	list=24%	signal=37%
	KEGG_LEISHMANIA_INFECTION	70	0.372054647	2.02425	0.00018	0.00147	0.0009	5569	tags=49%	list=24%	signal=37%
	KEGG_UBIQUITIN_MEDIATED_PROTEOLYSIS	131	0.300824615	1.89051	0.00022	0.00172	0.00105	6448	tags=50%	list=28%	signal=36%
	KEGG_LEUKOCYTE_TRANSENDOTHELIAL_MIGRATION	104	0.3117706	1.87525	0.00023	0.00173	0.00105	2876	tags=31%	list=12%	signal=27%
	KEGG_OOCYTE_MEIOSIS	103	0.31375048	1.87758	0.00024	0.00176	0.00107	6357	tags=47%	list=27%	signal=34%
	KEGG_RIG_I_LIKE_RECEPTOR_SIGNALING_PATHWAY	56	0.384820773	1.99106	0.00035	0.00249	0.00152	4393	tags=43%	list=19%	signal=35%
	KEGG_ERBB_SIGNALING_PATHWAY	82	0.333161159	1.86976	0.00043	0.0029	0.00177	6346	tags=50%	list=27%	signal=36%
	KEGG_PATHOGENIC_ESCHERICHIA_COLI_INFECTION	52	0.385477693	1.93389	0.00076	0.00496	0.00302	5806	tags=50%	list=25%	signal=38%
	KEGG_AMINOACYL_TRNA_BIOSYNTHESIS	22	-0.524674481	-2.09183	0.00101	0.00639	0.00389	4283	tags=50%	list=19%	signal=41%
	KEGG_ENDOMETRIAL_CANCER	49	0.384797916	1.89115	0.00121	0.00741	0.00452	6284	tags=57%	list=27%	signal=42%
	KEGG_FC_EPSILON_RI_SIGNALING_PATHWAY	71	0.329190718	1.7955	0.00127	0.00751	0.00458	4142	tags=41%	list=18%	signal=34%
	KEGG_REGULATION_OF_AUTOPHAGY	19	0.554313194	2.14975	0.00143	0.00817	0.00498	5583	tags=74%	list=24%	signal=56%
STIMATE	KEGG_PANCREATIC_CANCER	69	0.328134031	1.77954	0.00169	0.00938	0.00572	5671	tags=46%	list=24%	signal=35%
	KEGG_ALZHEIMERS_DISEASE	150	0.255766309	1.63223	0.00186	0.01003	0.00612	7370	tags=47%	list=32%	signal=32%
	KEGG_VIBRIO_CHOLERAE_INFECTION	49	0.369094082	1.81397	0.00205	0.01072	0.00653	3979	tags=41%	list=17%	signal=34%
	KEGG_ADIPOCYTOKINE_SIGNALING_PATHWAY	60	0.354929858	1.86447	0.00229	0.01142	0.00696	7386	tags=55%	list=32%	signal=38%
	KEGG_PROSTATE_CANCER	86	0.302260041	1.726	0.00234	0.01142	0.00696	6444	tags=50%	list=28%	signal=36%
	KEGG_NON_SMALL_CELL_LUNG_CANCER	52	0.360810135	1.81014	0.00237	0.01142	0.00696	5671	tags=50%	list=24%	signal=38%
	KEGG_FOCAL_ADHESION	191	0.237525975	1.59599	0.00265	0.01241	0.00757	5147	tags=35%	list=22%	signal=27%
	KEGG_CYTOSOLIC_DNA_SENSING_PATHWAY	40	0.39544046	1.83377	0.00299	0.01367	0.00833	4032	tags=45%	list=17%	signal=37%
	KEGG_SNARE_INTERACTIONS_IN_VESICULAR_TRANSPORT	38	0.392099474	1.8149	0.00318	0.01419	0.00865	5917	tags=47%	list=26%	signal=35%
	KEGG_COLORECTAL_CANCER	62	0.318658594	1.69119	0.00392	0.01629	0.00993	6479	tags=48%	list=28%	signal=35%
	KEGG_PURINE_METABOLISM	146	-0.24717968	-1.5779	0.00392	0.01629	0.00993	5510	tags=36%	list=24%	signal=28%
	KEGG_SPLICEOSOME	126	0.257261958	1.61085	0.00395	0.01629	0.00993	7532	tags=57%	list=33%	signal=39%
	KEGG_VALINE_LEUCINE_AND_ISOLEUCINE_DEGRADATION	42	-0.369687471	-1.76249	0.00401	0.01629	0.00993	5296	tags=45%	list=23%	signal=35%
	KEGG_ALANINE_ASPARTATE_AND_GLUTAMATE_METABOLISM	27	-0.435857502	-1.86222	0.0041	0.01629	0.00993	5510	tags=59%	list=24%	signal=45%
	KEGG_AMYOTROPHIC_LATERAL_SCLEROSIS_ALS	48	0.356152015	1.75201	0.00432	0.01683	0.01026	7265	tags=62%	list=31%	signal=43%
	KEGG_SULFUR_METABOLISM	13	0.580531759	2.01129	0.00462	0.01761	0.01074	6817	tags=69%	list=29%	signal=49%
	KEGG_ARGININE_AND_PROLINE_METABOLISM	50	-0.344237191	-1.70961	0.00542	0.01984	0.0121	6167	tags=50%	list=27%	signal=37%
	KEGG_HUNTINGTONS_DISEASE	165	0.234663202	1.53175	0.00542	0.01984	0.0121	7370	tags=45%	list=32%	signal=31%
	KEGG_LONG_TERM_POTENTIATION	60	0.331166183	1.73964	0.00735	0.02639	0.01609	5559	tags=47%	list=24%	signal=36%
	KEGG_GLIOMA	61	0.312760704	1.66332	0.0085	0.0299	0.01823	6444	tags=51%	list=28%	signal=37%
	KEGG_PPAR_SIGNALING_PATHWAY	59	0.316165767	1.64466	0.00892	0.03079	0.01877	5642	tags=37%	list=24%	signal=28%
	KEGG_COMPLEMENT_AND_COAGULATION_CASCADES	56	0.310959298	1.6089	0.01007	0.03414	0.02081	7158	tags=50%	list=31%	signal=35%
	KEGG_NOTCH_SIGNALING_PATHWAY	46	0.334088625	1.60909	0.01065	0.03542	0.0216	5322	tags=46%	list=23%	signal=35%
	KEGG_OXIDATIVE_PHOSPHORYLATION	108	0.246830851	1.48357	0.01172	0.03828	0.02334	6907	tags=45%	list=30%	signal=32%
	KEGG_N_GLYCAN_BIOSYNTHESIS	45	-0.340395481	-1.64496	0.01193	0.0383	0.02335	5130	tags=47%	list=22%	signal=36%
	KEGG_PROGESTERONE_MEDIATED_OOCYTE_MATURATION	81	0.274966319	1.54491	0.01238	0.03906	0.02382	6357	tags=44%	list=27%	signal=32%
	KEGG_VEGF_SIGNALING_PATHWAY	68	0.276265851	1.48678	0.01269	0.03935	0.024	6346	tags=46%	list=27%	signal=33%
	KEGG_PYRIMIDINE_METABOLISM	95	-0.263457308	-1.54023	0.01329	0.04054	0.02472	4682	tags=35%	list=20%	signal=28%
	KEGG_INTESTINAL_IMMUNE_NETWORK_FOR_IGA_PRODUCTION	44	-0.336623833	-1.60712	0.01608	0.04763	0.02904	6791	tags=52%	list=29%	signal=37%
	KEGG_SMALL_CELL_LUNG_CANCER	82	0.265420924	1.48959	0.01625	0.04763	0.02904	5671	tags=38%	list=24%	signal=29%
	KEGG_P53_SIGNALING_PATHWAY	66	0.294798529	1.57963	0.01678	0.04763	0.02904	2555	tags=29%	list=11%	signal=26%
	KEGG_BASAL_CELL_CARCINOMA	50	-0.310222669	-1.54068	0.0168	0.04763	0.02904	7885	tags=46%	list=34%	signal=30%
	KEGG_VASOPRESSIN_REGULATED_WATER_REABSORPTION	43	0.33424424	1.5993	0.01692	0.04763	0.02904	4182	tags=42%	list=18%	signal=34%

Table S9 Multiple linear regression analysis

Index	Estimate	Std. Error	t-value	p-value
(Intercept)	2.797546	2.314706	1.209	0.247
Age	0.002088	0.006249	0.334	0.743
APACHEII	-0.01607	0.007794	-2.062	0.058
diabetes.	-0.02884	0.168287	-0.171	0.866
spesis.	0.194138	0.163922	1.184	0.256
delirum.	-0.07264	0.171583	-0.423	0.678
A.CD14+CD16-	0.008517	0.01985	0.429	0.674
B.CD14+CD16-	0.001842	0.013775	0.134	0.896
C.CD14+CD16-	0.042964	0.048737	0.882	0.393
A.CD14loCD16+	-0.01444	0.013819	-1.045	0.314
B.CD14loCD16+	-0.00268	0.019016	-0.141	0.890
C.CD14loCD16+	0.00516	0.019871	0.26	0.799
A.IL6	-0.00057	0.001087	-0.525	0.608
B.IL6	-0.00616	0.00193	-3.194	0.006
C.IL6	0.000514	0.003168	0.162	0.873
A.TNFa	-0.0482	0.032629	-1.477	0.162
C.TNFa	0.086572	0.030422	2.846	0.013
C.SLC2A1	1.780568	0.883132	2.016	0.063
C.STIMATE	-3.43023	5.953605	-0.576	0.574
B.NECT%	-0.01387	0.028049	-0.495	0.629
C.NECT%	-0.01289	0.009933	-1.298	0.215
B.MONO	0.0784	0.208344	0.376	0.712
C.MONO	0.21432	0.203368	1.054	0.310
B.MONO%	-0.0753	0.047299	-1.592	0.134
C.MONO%	-0.07564	0.049863	-1.517	0.152
A.CRP	0.000941	0.001072	0.878	0.395
B.CRP	0.002162	0.002209	0.979	0.344
C.CRP	-0.00116	0.003355	-0.345	0.735
A.C3	-0.00096	0.000398	-2.422	0.030
B.C3	0.000687	0.000572	1.201	0.250

Adjusted R^2: 0.6794; p-value : 0.003578

Table S10 Primers of qPCR validation

Primer name	Sequence (5'→3')	Length	Location	Amplication Size (bp)	PrimerBank ID
GAPDH F	CTGGGCTACACTGAGCACC	19	694-712	101	378404907c3
GAPDH R	AAGTGGTCGTTGAGGGCAATG	21	794-774		
STIMATE F	CGGCGAATATGGAGACCCTC	20	417-436	152	38348401b3
STIMATE R	GGTTTTCAATGGGATTCAACAGG	23	568-546		
SLC2A1 F	ATTGGCTCCGGTATCGTCAAC	21	931-951	174	166795298c3
SLC2A1 R	GCTCAGATAGGACATCCAGGGTA	23	1104-1082		
